# Women's participation in the Paralympic Games (1960–2024): a longitudinal and bibliometric analysis of global trends and scientific production

**DOI:** 10.3389/fspor.2026.1834488

**Published:** 2026-09-30

**Authors:** Renato De Donato, Chiara Milanese, Nicole Lorenzet, Federico Schena, Valentina Cavedon

**Affiliations:** 1 Department of Neurosciences, Biomedicine and Movement Sciences, University of Verona, Verona, Italy; 2 National PhD Programme in Kinesiology and Sport Sciences, Department of Neurosciences, Biomedicine and Movement Sciences, University of Verona, Verona, Italy

**Keywords:** gender balance and equity, Paralympic athletes, Paralympic classification system, Paralympic sports, scientific production, women athletes

## Abstract

**Background:**

Women's participation in the Paralympic Games has expanded over time, yet this growth has been non-linear and uneven across regions, disciplines, and editions. At the same time, scientific research on women in Paralympic and parasports contexts remains limited and geographically concentrated. Studies that provide a comprehensive, long-term, sex-disaggregated reconstruction of participation across both Summer and Winter editions, together with an examination of its relationship to the geography of scientific production, are scarce.

**Methods:**

This study reconstructs sex-disaggregated participation in the Summer Paralympic Games (1960–2024) and Winter Paralympic Games (1976–2022) using official data provided by the International Paralympic Committee (IPC), harmonized with National Paralympic Committee data. Longitudinal trends and cross-national patterns are examined through descriptive and comparative analyses. Exploratory non-parametric analyses were used to assess longitudinal trends and macro-regional differences in demographic-normalized participation. As a complementary component, a systematic search of the Web of Science Core Collection (completed 31 October 2025) identified 71 relevant publications, which were analyzed using bibliometric techniques.

**Results:**

Women's participation in the Summer Paralympics follows a non-linear trajectory, reaching near parity at Paris 2024 (44.4%), whereas participation in the Winter Games remains consistently lower, with only modest long-term growth and no comparable movement toward parity (18%–25%). Two study-specific descriptive indicators, i.e., the Weighted Equity Index and the Normalized Female Participation Index, were used to summarize cross-national differences in internal delegation balance and demographic-normalized participation intensity. The bibliometric analysis documents a small but expanding body of literature that is geographically concentrated.

**Conclusion:**

These findings suggest a persistent misalignment between the increasing presence of women in the Paralympic Games and the comparatively limited and uneven development of scientific research on this population. The study provides a comprehensive longitudinal mapping of women's participation and offers an empirical basis for future research and policy aimed at improving equity in Paralympic sports.

## Introduction

From the inaugural Paralympic Games held in Rome in 1960 to the most recent Summer and Winter editions, women's participation in the Paralympic Movement has undergone a long-term transformation marked by a progressive—yet non-linear and uneven—expansion both numerically and geographically. The enlargement of the sports program, the increase in the number of participating National Paralympic Committees (NPCs), and the growing institutionalization of the Movement have all contributed, over the decades, to redefining the presence and role of women athletes with disabilities within the international sporting system (1–3). Despite these developments contributing to a gradual reduction of sex-based participation imbalances,^1^ particularly in the Summer Games, studies have shown that such growth has been neither linear nor uniformly distributed across geographical areas, sports disciplines, and national contexts (10–12).

In parallel, recent years have witnessed an increase in scientific research on gender equity in sports and on the lived experiences of women athletes in parasports, although the field remains characterized by relatively low publication volumes and a strong geographical concentration (13, 14). Overall, scientific production in the Paralympic domain is less substantial and less historically stratified than that concerning the Olympic field (15). Within this still emerging corpus, some document-based analyses have relied on International Paralympic Committee (IPC) archives to explore specific aspects of the Games' development, such as the evolution of women's participation in the “modern era” of the Summer Paralympics (1988–2024) (12) and trends in medal distribution across recent editions (1992–2016) (16). In particular, Santos et al. reconstructed the trajectory of women's participation in the Summer Paralympic Games from 1988 to 2024, focusing on regional patterns and the progressive movement toward equity in recent editions (12). However, these contributions remain either limited to descriptive indicators of participation or focused on performance outcomes, without providing a comprehensive, longitudinal, sex-disaggregated overview of the entire Paralympic trajectory (1960–present) or systematically linking participation patterns to the geography of scientific production.

Building on this literature, two gaps remain. First, the historical reconstruction of women's Paralympic participation is still incomplete across the full period from 1960 to the present, especially when the Summer and Winter editions are considered together. Santos et al. provide an important account of the modern Summer Paralympic period, but the early decades of the Movement and the Winter Games remain comparatively underexamined (12). Second, the growth of women's participation has rarely been examined alongside the development of scientific knowledge on women in Paralympic and parasports contexts. As a result, it remains unclear whether the increasing presence of women in the Games has been accompanied by a comparable expansion and diversification of scholarly attention.

To address these gaps, the present study aims to provide a comprehensive, longitudinal, sex-disaggregated reconstruction of the historical evolution of women's participation in the Summer and Winter Paralympic Games, covering the Summer editions from 1960 to 2024 and the Winter editions from 1976 to 2022, using official participation lists provided by the IPC. Such a reconstruction is necessary to assess whether the observed growth in women's participation reflects sustained structural change, episodic developments, or geographically uneven patterns across national contexts and types of Games. The analysis examines absolute numbers, participation percentages, and cross-national and macro-regional differences.

As a secondary objective, the study provides a bibliometric analysis of the international scientific literature explicitly focused on women in the Paralympic and parasports contexts, mapping its temporal growth, geographical distribution of affiliations, and primary publication venues (17).

The study is guided by the following four research questions: (a) How has women's participation changed across the Summer and Winter Paralympic Games over their respective historical trajectories? (b) Do these changes differ between Summer and Winter editions and across historical phases? (c) How is women's participation distributed across countries and geographical macro-areas when both absolute and demographic-normalized indicators are considered? (d) To what extent does the geography of scientific production on women in Paralympic and parasports contexts align with, or diverge from, the geography of women's participation in the Games? By addressing these questions, the study treats participation and scientific production as related but distinct dimensions: the first concerns the historical presence of women athletes in the Paralympic Games, whereas the second concerns the extent to which that presence has become an object of scientific research.

## Materials and methods

### Study design

This study adopts an observational, retrospective, descriptive–analytic design based on a mixed documentary–bibliometric approach, combining documentary analysis of archival sports data with a bibliometric analysis of scientific literature (18, 19).

 The documentary analysis draws on official participation data provided by the IPC, including all available editions of the Summer and Winter Paralympic Games from Rome 1960 to the most recent editions. The dataset enables a longitudinal, sex-disaggregated examination of participation, focusing on absolute numbers, percentage shares of women's participation per edition, and their distribution across National Paralympic Committees. The bibliometric analysis includes a systematic search of publications indexed in the Web of Science Core Collection relating to women in Paralympic and parasports contexts, followed by a bibliometric analysis conducted in R using the Biblioshiny interface (17). This approach enables structured mapping of the temporal growth, geographical distribution, and publication patterns of the field, and supports a comparative analysis of the relationship between the evolution of women's participation in the Paralympic Games and the development of corresponding scientific production over the same time span.

### Documentary sources and participation data

Information on participation in Paralympic events was obtained exclusively from the official website of the IPC, using the historical sections detailing the results and start lists/participation lists for each edition of the Summer and Winter Paralympic Games. All official editions for which complete or reconstructable sex-disaggregated data were available were included, while youth events, world championships, continental tournaments, qualifying events, and non-medal competitions were excluded. At the country level, only NPCs officially recognized by the IPC and with at least one accredited athlete in the respective editions were considered.

For each edition, the dataset incorporated the sex attribution of all accredited athletes and their national affiliation. The variables included in the harmonized database therefore comprised the number of women and men athletes per edition (both overall and disaggregated by NPC) and delegation composition by sex for each country–edition combination.

Prior to analysis, the dataset underwent a structured cleaning procedure. This involved checking internal consistency (including the sum of men and women vs. official totals), identifying and removing duplicate entries, and verifying national attributions in cases of changing country denominations or geopolitical reconfigurations (e.g., state unifications or separations), in line with established practices in cross-national and longitudinal data harmonization (20). Missing values that could not be reconstructed reliably were marked as missing and excluded from analyses requiring complete data, without applying imputation procedures, following standard recommendations in the methodological literature on missing data (21, 22). To quantify the extent of this issue, we examined, for each NPC, the proportion of missing edition-level records falling within that NPC's active participation window—that is, between its first and last recorded appearance in the respective Games—thereby excluding editions preceding an NPC's entry into the Movement or following its withdrawal, which do not represent missing data in a strict sense. Within this active window, missing records accounted for 8.8% of NPC-by-edition cells in the Summer Games and 18.1% in the Winter Games. Missing records were markedly more frequent in earlier editions than in recent ones (Summer Games: 14.6% for 1960–1988 vs. 7.2% for 1992–2024; Winter Games: 25.0% for 1976–1988 vs. 15.9% for 1992–2022), consistent with the lower documentation standards of the earliest editions of the Movement. This active-window approach should be regarded as an upper-bound estimate of genuine documentation gaps, since it cannot fully distinguish undocumented participation from legitimate, occasional non-participation—for financial, political, or logistical reasons—by NPCs that otherwise remained active in the Movement. These procedures produced a harmonized, sex-disaggregated dataset suitable for longitudinal and cross-national analyses of participation and delegation composition.

#### Geographical macro-area classification

To analyze large-scale geographical patterns in Paralympic participation, all countries were grouped into geographical macro-areas. The classification adopted in this study follows a mixed geopolitical–regional criterion, informed primarily by the regional framework of the World Bank Data (23). Accordingly, each country was assigned to one of the following macro-areas: Western Europe, Eastern Europe/Former USSR, North America, Latin America, the Asia-Pacific, Africa and the Middle East, and Special Categories (Unified Team, Independent Paralympic Participants, Individual Paralympic Athletes, and Neutral Paralympic Athletes). Every National Paralympic Committee appearing in the participation dataset was assigned to a macro-area. Historically dissolved national teams (Czechoslovakia, East Germany, West Germany, Yugoslavia, Serbia and Montenegro, and Rhodesia) were assigned to the macro-area corresponding to their historical geographic location, rather than left unclassified or grouped with the Special Categories, which are reserved for the four IPC non-national participation categories listed above.

This categorization was applied consistently across all editions of the Paralympic Games and enabled the aggregation of country-level data into broader regional blocs, facilitating the analysis of large-scale geographical differences in sex-disaggregated participation (Table 1).

**Table 1:** The cumulative number of female athletes participating in all Summer (1960–2024) and Winter (1976–2022) Paralympic Games, aggregated by geographical macro-area according to the regional classification described in the methods section.

Geographical macro-area	Total number of female athletes—Summer Games (1960–2024)	Total number of female athletes—Winter Games (1976–2022)
Western Europe	6,012	687
Eastern Europe/Former USSR	749	147
North America	1,938	265
Latin America	1,344	3
The Asia-Pacific	2,960	142
Africa and the Middle East	1,293	5
Special categories	56	23

Special categories include the four official IPC non-national categories (Unified Team, Independent Paralympic Participants, Individual Paralympic Athletes, and Neutral Para Athlete); historically dissolved national teams (Czechoslovakia, East Germany, West Germany, Yugoslavia, Serbia and Montenegro, and Rhodesia) are assigned to their corresponding geographical macro-area.

### Bibliometric sources

#### Databases and software

For the bibliometric analysis, the Web of Science Core Collection (Clarivate) was used as the sole data source, which was selected as it is a generalist database with broad coverage and indexing standards widely adopted for the evaluation of scientific output. All relevant records were exported from Web of Science in a text format compatible with the R environment and imported into a dedicated project. The analysis was then conducted in R (version 4.4.2; R Foundation for Statistical Computing) using the *Bibliometrix* package and its web interface, *Biblioshiny* (17). These tools were employed for corpus management (deduplication and harmonization of bibliographic fields), calculation of bibliometric indicators (e.g., productivity by year and country), and generation of graphical visualizations.

#### Search strategy, inclusion/exclusion criteria, and study selection process

The literature search was conducted in the Web of Science (Clarivate) database using a structured and reproducible search string designed to capture both the Paralympic Movement and the various parasports disciplines, with an explicit focus on women. Web of Science was selected due to its high indexing standards, broad coverage of peer-reviewed journals, and its widespread use in bibliometric studies (18). The search strategy combined, in the Topic (TS) and Abstract (AB) fields, terms referring to the Paralympic Games and Paralympic sports in general and to individual disciplines.

This first component was combined, using the Boolean operator AND, with specific descriptors relating to women (TS = female OR woman OR women). These terms were included to ensure comprehensive coverage of both sex-based and gender-related terminology in the indexed literature. The query covered the full time span indexed by Web of Science for this topic, and eligible publications fall within the period 1999–2025 (last search: 31 October 2025). The full search string strategy is reported in Table 2.

**Table 2:** Search string strategy.

Full String Strategy
(TS =(“Paralympic Game*” OR “Summer Paralympic Game*” OR “Winter Paralympic Game*” OR Paralympic* OR Parasport* OR Para-sport* OR “Para sport*”) OR (TS = (“Para-Athlete*” OR “Para athlete*” OR Parathlete* OR “Paralympian*” OR “Paralympic athlete*” OR “Paralympic competitor*” OR “Paralympic player*” OR “Paralympic sportsm*” OR “Paralympic sportswom*” OR “Paralympic cyclist*” OR “Para swimmer*” OR “Paralympic swimmer*” OR “Para sprinter*” OR “Paralympic sprinter*” OR “Goalball player*” OR “Sitting volleyball player*” OR “Wheelchair tennis player*” OR “Wheelchair basketball player*” OR “Wheelchair rugby player*” OR “Boccia player*” OR “Para athletics competitor*”))
OR AB = (“Para Athletic*” OR “Para Badminton*” OR “bocc*” OR “Para Rowing*” OR “Para Canoe*” OR “Para Road Cycling*” OR “Para Track Cycling*” OR “Para Equestrian*” OR “Football 5-a-side*” OR “goalwill*” OR “Para Judo*” OR “Para Swimming*” OR “Para Powerlifting*” OR “Wheelchair Rugb*” OR “Wheelchair Fencing*” OR “Sitting Volleyball*” OR “Para Taekwondo*” OR “Wheelchair Tennis*” OR “Para Table Tennis*” OR “Para Shooting*” OR “Para Archery*” OR “Para Triathlon*” OR “Wheelchair Basketball 3 × 3*” OR “Bowling*” OR “Football 7-a-side*” OR “Wheelchair Slalom*” OR “Para Sailing*” OR “Para Biathlon*” OR “Wheelchair Curling*” OR “Para Ice Hockey*” OR “Para Alpine Skiing*” OR “Para Nordic Skiing*” OR “Para Snowboard*” OR “Para Luge*” OR “Wheelchair Sport*” OR “Wheelchair-Sport*” OR “Wheelchair Court Sport*” OR “Wheelchair-Court-Sport*” OR “Para-Athletic*” OR “Para-Badminton*” OR “bocc*” OR “Para-Rowing*” OR “Para-Canoe*” OR “Para-Road-Cycling*” OR “Para-Track-Cycling*” OR “Para-Equestrian*” OR “Football-5-a-side*” OR “goalwill*” OR “Para-Judo*” OR “Para-Swimming*” OR “Para-Powerlifting*” OR “Wheelchair-Rugb*” OR “Wheelchair-Fencing*” OR “Sitting-Volleyball*” OR “Para-Taekwondo*” OR “Wheelchair-Tennis*” OR “Para-Table Tennis*” OR “Para-Shooting*” OR “Para-Archery*” OR “Para-Triathlon*” OR “Wheelchair-Basketball 3 × 3*” OR “Wheelchair-Dance-Sport*” OR “Football-7-a-side*” OR “Wheelchair-Slalom*” OR “Para-Sailing*” OR “Para-Biathlon*” OR “Wheelchair-Curling*” OR “Para-Ice-Hockey*” OR “Para-Alpine-Skiing*” OR “Para-Nordic-Skiing*” OR “Para-Snowboard*”)) AND TS = (female* OR woman* OR women*)

To further refine the record set and reduce non-relevant literature, automatic exclusion filters were applied to document types and Web of Science categories. Proceedings papers, book chapters, book reviews, retracted publications, biographical items, editorial materials, and meeting abstracts were excluded, as they are not consistently subject to full peer-review processes. Thematic categories clearly unrelated to the object of the study (e.g., literature and philology, arts, music, theatre studies, certain areas of the natural and engineering sciences not connected to sports or movement sciences, zoology, marine and freshwater biology, and archaeological and paleontological disciplines) were likewise excluded using Web of Science Category filters. Language filters were also applied, retaining only articles written in English.

Only original research articles published in peer-reviewed scientific journals, in English, that specifically addressed women-related topics in Paralympic or parasports contexts (for example, studies on participation, performance, health, classification, or the experiences of Paralympic women athletes) were considered eligible. The following were excluded: (i) all contributions classifiable as grey literature (conference proceedings, technical reports, editorials, commentaries, biographical notes, etc.), even when indexed; (ii) studies with mixed samples in which the female dimension did not constitute a specific and distinct object of analysis (e.g., studies in which women and men are aggregated without distinction and it is not possible to extract separate results or discussions for women athletes); and (iii) articles referring generically to disability or adapted sports without a clear reference to the Paralympic or parasports context and without an explicit focus on the female sex.

The article selection process followed a standardized and documented procedure, in accordance with the Preferred Reporting Items for Systematic reviews and Meta-Analyses (PRISMA) 2020 guidelines for transparent reporting of literature selection processes (24). After exporting the results from Web of Science, the identification phase yielded 787 records in total. Application of the automatic filters described above led to the preliminary removal of 273 clearly ineligible records, leaving 514 records for screening. This set then underwent title and abstract screening by two independent reviewers, using Rayyan (25) to support blinded assessment and conflict resolution; the reviewers assessed adherence to the predefined inclusion/exclusion criteria.

In this phase, 442 records were excluded due to lack of thematic relevance, exclusive focus on male subjects, absence of references to the Paralympic context, or absence of any gender/sex analysis. The remaining 72 contributions were selected for full-text assessment. During this phase, one article that had initially met the inclusion criteria was excluded because it had been formally retracted from publication, leaving 71 articles for full reading (Figure 1).

**Figure 1:**
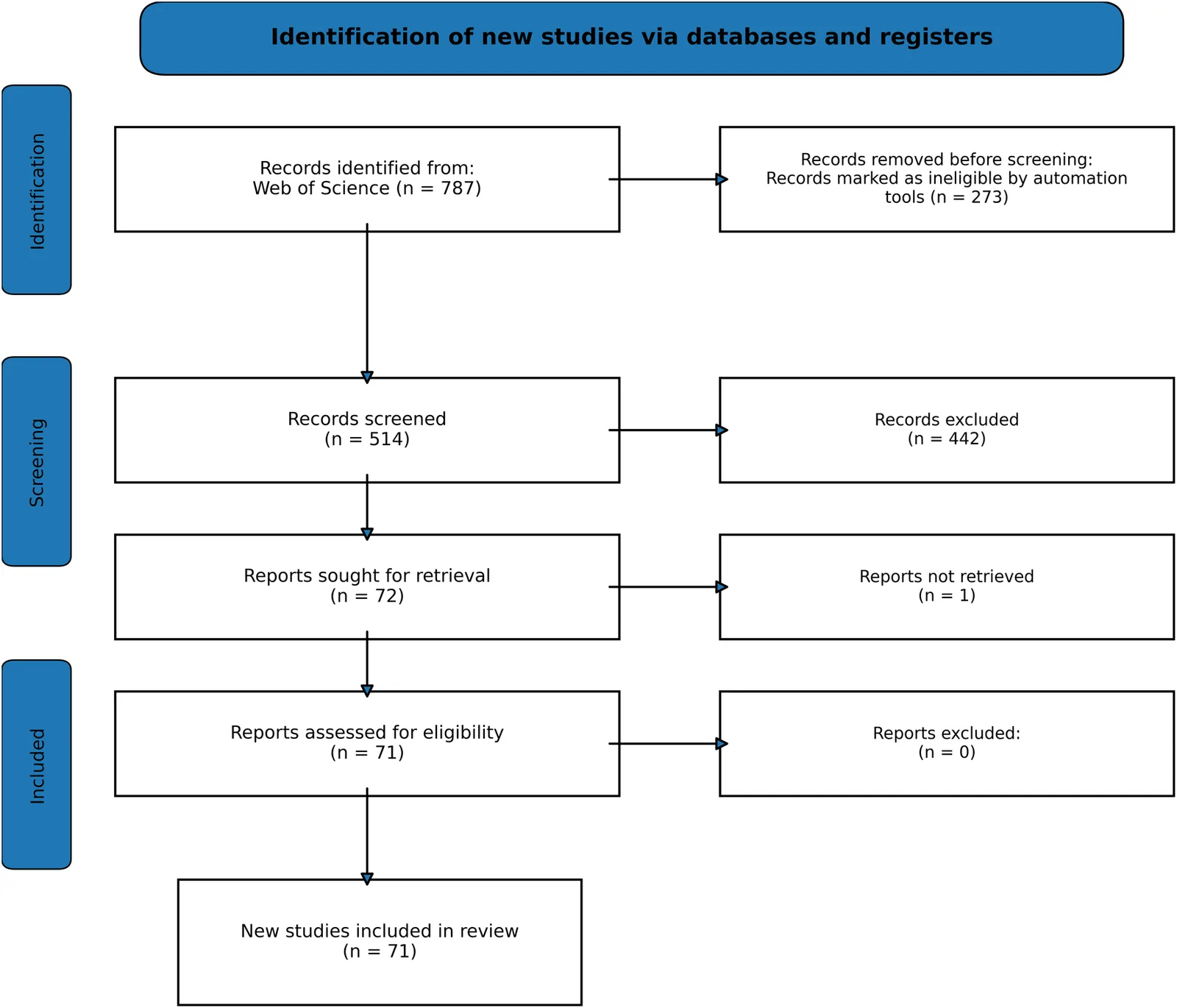
Flowchart of the literature search and review.

Full-text assessment was again conducted independently by two reviewers; in the event of disagreement regarding the inclusion of a study, a third senior reviewer intervened to reach a reasoned consensus on the final classification (inclusion/exclusion). At the end of this process, the final sample for the bibliometric analysis consisted of 71 articles, all centered on women-related topics in the context of the Paralympic Games or parasports disciplines. The complete workflow, comprising identification, screening, eligibility, and inclusion, is summarized in the PRISMA diagram and constitutes the documentary basis for the bibliometric analyses subsequently conducted in R via the Biblioshiny interface.

#### Bibliometric analysis

The bibliometric analysis was based on the final dataset of 71 articles and aimed to reconstruct the main structural features of scientific production on women in Paralympic sports. From this corpus, a set of descriptive indicators was extracted, including the publication time span, total number of documents, and annual growth rate. Temporal trends in scientific production were examined by calculating the annual number of publications and modeling the resulting trajectories to identify phases of acceleration, stability, or stagnation in the field. At the geographical level, country-specific scientific output was assessed by determining the number of publications associated with authors' institutional affiliations.

These bibliometric results provided the analytical foundation for subsequent comparisons with sex-disaggregated participation data from the Paralympic Games, enabling the exploration of convergences and divergences between competitive participation and scholarly attention.

### Data analysis

The analysis of the data consisted of two main components: (a) the processing of documentary data on participation in the Paralympic Games (1960–2024 for the Summer editions; 1976–2022 for the Winter editions) and (b) the bibliometric analysis of the selected scientific corpus (1999–2025). Analytical procedures followed a descriptive–comparative approach aimed at reconstructing the relationship between female participation and scientific production.

#### Analysis of participation data

The harmonized participation dataset was processed using Microsoft Excel to generate the primary analytical tables and to compute the sex-disaggregated indicators required for the longitudinal assessment of female representation across Paralympic editions. Standard spreadsheet functions were used to derive the proportional measures employed in the analysis, while ensuring coherence with the dataset structure described above in *Documentary sources and participation data*.

To analyze the evolution of internal sex equity, that is, the proportion of women within each national delegation for every edition, the time series of individual NPCs were subsequently processed using MATLAB (The MathWorks, Inc., MA, USA). This software enabled management of discontinuous national trajectories, handling of structurally fragmented sequences, and generation of chronological profiles. For each NPC, values across editions were consolidated into continuous historical series, which served to identify differentiated patterns of internal equity development (e.g., early and sustained increases, delayed growth, intermittent participation, and stagnating trajectories).

#### Exploratory statistical analysis of longitudinal trends

To support the descriptive interpretation of longitudinal participation patterns, exploratory non-parametric statistical analyses were conducted. Spearman's rank correlation was used to assess the association between year of edition and female participation percentage separately for the Summer and Winter Paralympic Games. Sen's slope was additionally calculated as a robust estimate of the average temporal change in female participation percentage.

Differences in female participation percentages between the Summer and Winter editions were explored using the Mann–Whitney *U*-test. For the Summer Games, editions were also grouped into three historically informed phases, namely, early expansion (1960–1976), stagnation/fluctuation (1980–2000), and recent acceleration (2004–2024), and differences across the phases were examined using the Kruskal–Wallis test. These analyses were interpreted as exploratory and non-causal, in line with the retrospective and descriptive–analytic design of the study.

#### Calculation of the Weighted Equity Index

To complement edition-level participation percentages, two study-specific indicators were used in this analysis: the Weighted Equity Index (WEI) and the Normalized Female Participation Index (NFP). Both were designed as transparent descriptive tools for this retrospective analysis, following the general principle that composite and normalized indicators should be explicitly defined, reproducible, and interpreted in relation to their underlying assumptions (26). They are not presented as externally validated measures of gender equity, but as operational summaries intended to support cross-national and longitudinal comparison. The first indicator, the WEI, summarizes the average internal sex composition of each NPC delegation across the editions in which that NPC participated.

Let i denote a Paralympic edition (i=1,…,T) and let $E_{i}\;$ be the proportion of women in the delegation of country *c* in edition *i*. Let $w_{i}$ be an indicator weighting the presence of the NPC in edition *i*, defined as

$$w_{i}=\{\begin{array}{cc} 1, & \mathrm{if}\;\mathrm{the}\;\mathrm{NPC}\;\mathrm{participated}\;\mathrm{in}\;\mathrm{edition}\;i,\\ 0, & \mathrm{otherwise}. \end{array}$$

The WEI for country *c* is then computed as a weighted average, as follows:

$$\mathrm{WE}I_{c}=\frac{\sum_{i=1}^{T}w_{i}E_{i}}{\sum_{i=1}^{T}w_{i}}.$$

This formulation assigns equal weight to each edition in which the NPC participated and excludes non-participating editions from the denominator. The index is therefore directly interpretable as the mean female share of an NPC's delegation across its participating editions. It ranges from 0 to 1, where higher values indicate a higher average proportion of women within that NPC's delegations over time.

The WEI should not be interpreted as a direct measure of all dimensions of gender equity, nor as a measure of access, investment, policy effectiveness, or athlete development pathways. It captures only one dimension of internal delegation composition: the average proportion of women among accredited athletes in the editions in which the NPC competed. Participation continuity was examined separately through the number of editions in which each NPC fielded women athletes.

To clarify the computation, consider a hypothetical NPC participating in two editions. In Edition 1, the delegation includes four athletes, of whom two are women $\left(E_{1}=2/4=0.50\right)$. In Edition 2, the delegation includes 10 athletes, of whom 5 are women $\left(E_{2}=5/10=0.50\right)$. Since the NPC participates in both editions $\left(w_{1}=w_{2}=1\right)$, the WEI is simply the average of the two equity values, as follows:

$$\mathrm{WEI}=\frac{E_{1}+E_{2}}{2}=\frac{0.50+0.50}{2}=0.50.$$

This example illustrates that the WEI corresponds to the arithmetic mean of the proportion of female athletes at the delegation level across all editions in which the NPC has competed, thereby offering a transparent and replicable descriptive summary of long-term internal delegation balance.

Like any average computed over a variable number of editions, the WEI is sensitive to the number of editions on which it is based. For NPCs that only participated in a few editions, the WEI reduces to the mean of a small number of edition-level values, so a single edition with an unusually high or low female participation can shift the country-level index disproportionately relative to NPCs with longer, more stable participation histories. This instability is a general property of averages computed over small samples, rather than an artifact of the present calculation. For this reason, WEI values should always be interpreted alongside the number of editions on which they are based (reported alongside WEI in Table 3), and comparisons involving NPCs that participated in a few editions should be treated with particular caution.

**Table 3:** Female participation in the Summer Paralympic Games (1960–2024): percentage of women and total number of female and male athletes by edition.

Edition	Female participation (%)	Total number of female athletes	Total number of male athletes
Rome 1960	21.5	45	164
Tokyo 1964	26.7	71	195
Tel Aviv 1968	25.3	196	578
Heidelberg 1972	29.0	268	653
Toronto 1976	21.5	271	1,000
Arnhem 1980	25.9	428	1,225
Stoke Mandeville & New York 1984	25.5	536	1,569
Seoul 1988	22.1	671	2,371
Barcelona 1992	23.3	699	2,300
Atlanta 1996	24.3	790	2,462
Sydney 2000	25.5	987	2,883
Athens 2004	30.6	1,149	2,600
Beijing 2008	34.6	1,367	2,585
London 2012	35.4	1,502	2,741
Rio 2016	38.6	1,671	2,657
Tokyo 2020	42.0	1,846	2,547
Paris 2024	44.4	1,970	2,463

#### Normalized Female Participation Index

The second study-specific indicator was the NFP, a demographic-normalized participation rate. Its aim was to reduce the influence of national population size on cross-country comparisons by expressing the average number of women athletes fielded per edition relative to the female population aged 15–64.

Let

*W*_tot_ = total number of female athletes entered by country *c* across all Paralympic editions;
*N*_ed_ = number of editions in which the NPC participated;
*FemPop*_15–64_ = average female population aged 15–64 (World Bank data) across the corresponding historical period.

Thus, the NFP is defined as follows:

$$\mathrm{NF}P_{c}=\frac{W_{\mathrm{tot}}}{N_{\mathrm{ed}}\times FemPop_{15\text{–}64}}.$$

This formulation yields the average number of women athletes per edition, normalized by the size of the female population aged 15–64. It therefore provides a demographic-adjusted perspective on women's Paralympic participation, complementing both absolute participation counts and delegation-level proportions of female participants.

A theoretically preferable denominator for the NFP would be the national population of women with disabilities, as this group represents the demographic base most directly linked to Paralympic eligibility. However, globally comparable datasets disaggregated by sex, age group, and disability status are not available for the majority of countries and time periods covered in this study. Existing data show marked heterogeneity in definitions of disability, survey instruments, reporting frequency, and statistical coverage, with many countries lacking reliable or up-to-date information altogether. These inconsistencies preclude the construction of a standardized disability-specific denominator suitable for longitudinal and cross-national comparison.

For this reason, the NFP adopts the female population aged 15–64 as a proxy denominator, in line with established conventions in demographic modeling and international comparative research (27) While this approach does not capture variation in disability prevalence among women, it allows the index to provide a standardized demographic denominator across countries and historical periods. The NFP should therefore be interpreted as a demographic-normalized participation rate, not as a direct measure of national inclusion policy, access to parasports systems, or the actual population eligible for Paralympic sports.

Because the NFP raw values are typically very small $\left(10^{-4}-10^{-9}\right)$, all values were multiplied by 100,000 to improve readability, following established epidemiological conventions for rate scaling (28). The rescaled NFP was used in all figures and comparative rankings.

Thus, for a hypothetical NPC that has entered a total of 30 women across five editions of the Paralympic Games, with an average female population aged 15–64 of 2,000,000, the NFP is computed as follows:

$$\mathrm{NFP}=\frac{30}{5\times 2,000,000}=\frac{30}{10,000,000}=3.0\times 10^{-6}.$$

After rescaling by 100,000, the value is as follows:

$$\mathrm{NF}P_{\mathrm{rescaled}}=3.0\times 10^{-6}\times 100,000=0.30.$$

This value expresses the average number of women athletes entered per edition per 100,000 women aged 15–64 in the national population. It provides a demographic-normalized indicator of participation intensity and should be read alongside absolute counts, number of editions, and delegation-level proportions of female participants.

As with any population-normalized rate, the NFP is sensitive to small numerators and small population denominators. For National Paralympic Committees with a very small female population aged 15–64 (e.g., island states and microstates) or with only a handful of women athletes entered across their participating editions, a single edition can produce disproportionately high NFP values relative to countries with larger, more stable delegations. This instability is a well-documented property of rate-based indicators applied to small populations (28), rather than an artifact of the present calculation. For this reason, NFP rankings should always be interpreted alongside the underlying raw counts (women entered and number of editions, reported alongside NFP in Tables 4, 5), and conclusions drawn from the highest-ranked countries should be treated with particular caution when these counts are small.

**Table 4:** Summary indicators for normalized female participation in the Summer Paralympic Games.

Country	Women_total	N_editions	FemPop_15_64	NFP corretto × 100.000 (=W_tot/(N_ed × FemPop) × 100.000)
Faroe Islands	13	10	16,124	8.06
Bermuda	11	8	20,676	6.65
Liechtenstein	2	4	13,069	3.83
Grenada	3	2	39,432	3.80
Iceland	35	12	129,886	2.25
Tonga	4	6	33,102	2.01
US Virgin Islands	2	4	32,751	1.53
Bahamas	8	5	146,848	1.09
Slovenia	61	9	646,328	1.05
Samoa	3	5	60,656	0.99
Estonia	28	9	427,192	0.73
Maldives	2	2	140,853	0.71
Ireland	191	17	17,76,289	0.63
Malta	12	11	177,686	0.61
Norway	172	16	17,72,144	0.61

The table reports, for each NPC, the cumulative historical number of women who entered the Summer Paralympic Games (Women_total), the number of editions in which the NPC fielded female athletes (N_editions), and the corresponding female population aged 15–64 years (FemPop_15–64). These variables are used to calculate the Summer Games NFP, shown both in its raw form and rescaled per 100,000 female inhabitants to allow cross-national comparison of demographically adjusted women's participation.

**Table 5:** Summary indicators for normalized female participation in the Winter Paralympic Games.

Country	Women_total	N_editions	FemPop_15_64	Winter Games normalized female participation × 100.000
Liechtenstein	1	3	13,069	2.55
Andorra	2	6	28,623	1.16
Norway	80	13	17,72,144	0.35
Estonia	7	5	427,192	0.33
Iceland	2	5	129,886	0.31
Finland	57	13	16,97,396	0.26
Austria	88	13	29,65,352	0.23
Slovakia	24	8	17,60,413	0.17
Switzerland	51	13	29,01,135	0.14
Sweden	47	13	32,09,212	0.11
Lithuania	1	1	936,532	0.11
Belarus	21	7	30,89,360	0.10
Armenia	6	6	10,80,901	0.09
Bosnia and Herzegovina	3	4	10,24,823	0.07
Denmark	14	12	18,77,887	0.06

The table reports, for each NPC, the cumulative historical number of women who entered the Winter Paralympic Games (Women_total), the number of editions in which the NPC fielded female athletes (N_editions), and the corresponding female population aged 15–64 years (FemPop_15–64). These variables are used to calculate the Winter Games NFP, shown both in its raw form and rescaled per 100,000 female inhabitants to allow cross-national comparison of demographically adjusted women's participation.

#### Regional comparison of normalized female participation

To examine whether demographic-normalized female participation differed across geographical macro-areas, country-level NFP values were compared across regions separately for the Summer and Winter Paralympic Games using the Kruskal–Wallis test. The rescaled NFP per 100,000 female inhabitants was used as the dependent variable. A non-parametric approach was adopted because the NFP values were highly skewed and group sizes differed substantially across macro-areas. When the overall Kruskal–Wallis test was significant, pairwise *post hoc* comparisons were conducted using Dunn's test with Holm correction for multiple comparisons. NFP values equal to zero were retained when present, in order to avoid upwardly biasing regional estimates. Special categories not corresponding to a national population denominator were not included in this analysis.

#### Bibliometric analysis procedures

The bibliometric analyses were conducted entirely using *Biblioshiny*, the web interface of the *Bibliometrix* package in R, which enabled the systematic processing of all records obtained through the selection procedure described above in *Search strategy, inclusion/exclusion criteria, and study selection process*.

Using the platform's standard analytical modules, we generated descriptive indicators and computed reconstructed country-level scientific production*. Biblioshiny*'s visualization tools were used to produce maps and graphs illustrating geographical distribution and temporal evolution within the corpus. All analyses were carried out using default or standardized parameters, guaranteeing replicability and methodological comparability across outputs.

#### Integrated analysis of participation and scientific production

The relationship between female participation and scientific production was examined by integrating each country's WEI value with its total publication count. To facilitate comparability between these two dimensions, a median-based normalization was applied to both the distribution of WEI scores and the distribution of scientific output. The two medians served as reference thresholds for constructing a two-by-two analytical matrix, in which each country was assigned to one of four quadrants (High/Low WEI × High/Low Scientific Production). This graphical and classificatory procedure enabled the identification of empirical patterns of convergence or divergence between WEI values and scholarly activity, distinguishing (a) contexts with both high WEI values and high scientific production; (b) contexts with substantial scientific output but medium or low WEI values; (c) contexts characterized by high WEI values but limited scholarly production; and (d) contexts displaying low values in both dimensions.

## Results

### Female participation in the Paralympic Games

#### Historical trends—Summer Games

Based on the dataset reconstructed from the IPC participation lists, the number and share of female athletes at the Summer Paralympic Games increased markedly over the period from Rome 1960 to Paris 2024. As summarized in Table 3 (absolute numbers of male and female athletes and percentage of female participation by edition), women accounted for roughly one-fifth of all athletes in the earliest Games (approximately 21.5% in Rome 1960). Their proportion grew during the late 1960s and early 1970s, then declined and remained at relatively modest and fluctuating levels through the late 1970s, 1980s, and 1990s, generally stabilizing around 22%–26%.

From the early 2000s onwards, the proportion of female participants had a more sustained upward trajectory. Following Sydney 2000 and Athens 2004, the proportion of women increased in each subsequent edition (Beijing 2008, London 2012, Rio 2016, and Tokyo 2020), reaching approximately 44.4% in Paris 2024, where women represented just under half of all accredited athletes. Overall, the historical pattern reveals a first phase of growth (1960s–early 1970s), a phase of stagnation and fluctuation at relatively low levels (late 1970s–1990s), and a phase of accelerated growth from the 2000s onwards, approaching numerical parity in the most recent editions.

#### Historical trends—Winter Games

Since their inception in Örnsköldsvik 1976, the Winter Paralympic Games exhibit a markedly different temporal trajectory of female participation compared with the Summer Games. As reported in Table 6, the proportion of women participants remained within a relatively narrow interval throughout the 1976–2022 period, generally fluctuating between approximately 18% and 24%–25%. Although the absolute number of female athletes increased over time, following the overall expansion of the Games, the percentage of women remained structurally lower than in the Summer Paralympic Games.

**Table 6:** Female participation in the Winter Paralympic Games (1976–2022): percentage of women and total number of female and male athletes by edition.

Edition	Female participation (%)	Total number of female athletes	Total number of male athletes
Ornskoldsvik 1976	18.7	37	161
Geilo 1980	23.4	70	229
Innsbruck 1984	22.4	94	325
Innsbruck 1988	20.4	77	300
Albertville 1992	21.1	77	288
Lillehammer 1994	19.2	90	379
Nagano 1998	21.7	122	440
Salt Lake City 2002	21.0	87	328
Torino 2006	20.9	99	375
Vancouver 2010	24.1	121	382
Sochi 2014	23.9	129	411
Pyeongchang 2018	23.6	133	431
Beijing 2022	24.4	136	422

Across the full historical series, the long-term increase is limited, and recent editions do not show movement toward numerical parity. Taken together, the data indicate a stable pattern in which women consistently account for roughly one-fifth to one-quarter of all accredited athletes at the Winter Paralympic Games.

#### Longitudinal trend analysis of female participation

The exploratory trend analysis confirmed a positive association between year of edition and female participation percentage in both the Summer and Winter Paralympic Games, although with markedly different magnitudes (Table 7; Figure 2). In the Summer Games, female participation increased significantly over time (Spearman's *ρ* = 0.716, *p* = 0.0012), with Sen's slope indicating an average increase of 0.296 percentage points per year, corresponding to approximately 2.96 percentage points per decade (Table 7).

**Table 7:** Exploratory statistical analysis of longitudinal female participation trends in the Summer and Winter Paralympic Games.

Games	*N* editions	Period	Spearman's *ρ*	*p* value	Sen's slope, pp/year	Sen's slope, pp/decade	Sen's slope, pp/4-year cycle
Summer Paralympic Games	17	1960–2024	0.716	0.0012	0.296	2.964	1.186
Winter Paralympic Games	13	1976–2022	0.632	0.0205	0.095	0.946	0.379

Spearman's rank correlation was used to assess the association between edition year and female participation percentage. Sen's slope estimates the average temporal change in female participation percentage, expressed in percentage points (pp). The distribution of female participation percentages differed significantly between the Summer and Winter editions, Mann–Whitney *U* = 26.00, *z* = −3.536, *p* = 0.0004. For the Summer Games, differences across the three historical phases were also significant [Kruskal–Wallis H(2) = 11.00, *p* = 0.0041].

**Figure 2:**
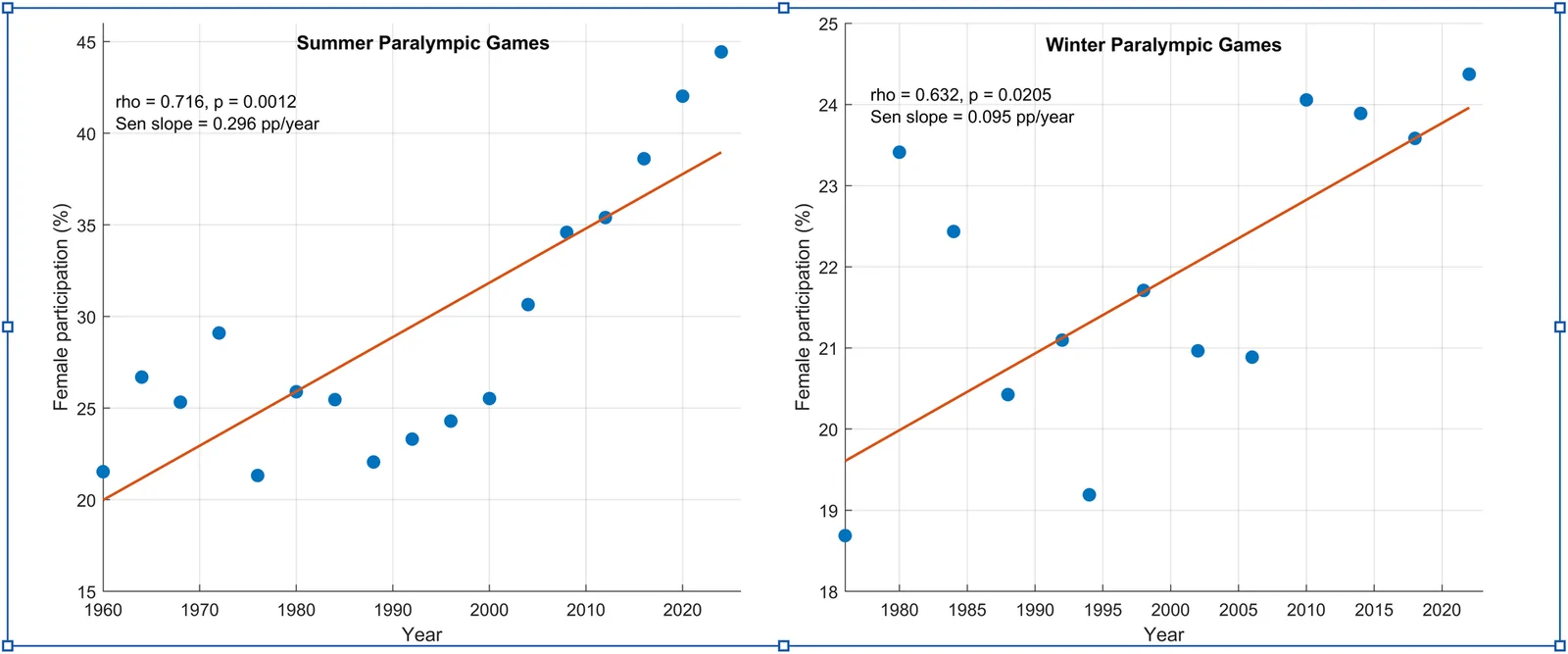
Longitudinal trends in women's participation in the Summer and Winter Paralympic Games. The figure shows the proportion of women athletes by edition year for the Summer Games (1960–2024) and Winter Games (1976–2022). Trend lines are based on Sen's slope estimates, with Spearman's correlation coefficients reported for each series.

In the Winter Games, the association between year and female participation percentage was also positive and statistically significant (Spearman's *ρ* = 0.632, *p* = 0.0205), but the estimated slope was substantially smaller, at 0.095 percentage points per year, corresponding to approximately 0.95 percentage points per decade (Table 7). Thus, while both series show a long-term increase, the Summer Games display a much stronger trajectory toward parity, whereas the Winter Games remain within a persistently lower participation range (Figure 2).

The distribution of female participation percentages differed significantly between the Summer and Winter editions, as assessed by the Mann–Whitney *U*-test (Table 7). In addition, the Kruskal–Wallis test indicated significant differences across the three historical phases of the Summer Games, supporting the descriptive periodization into early expansion, stagnation/fluctuation, and recent acceleration (Table 7).

### Distribution by country—Summer Games

The disaggregated analysis by NPC shows substantial heterogeneity in cumulative volumes of women's participation. In absolute terms, a limited group of NPCs with long-standing engagement in the Paralympic movement accounts for the highest historical totals. The United States of America occupies the leading position, followed by Canada, Austria, and Norway. Relatively high cumulative participation is also observed for Japan, the Russian Federation, and Germany. Intermediate levels characterize Finland, Switzerland, and Sweden, while Ukraine, France, the People's Republic of China, Italy, and Poland display lower but still non-negligible cumulative volumes within the overall distribution (Figure 3).

**Figure 3:**
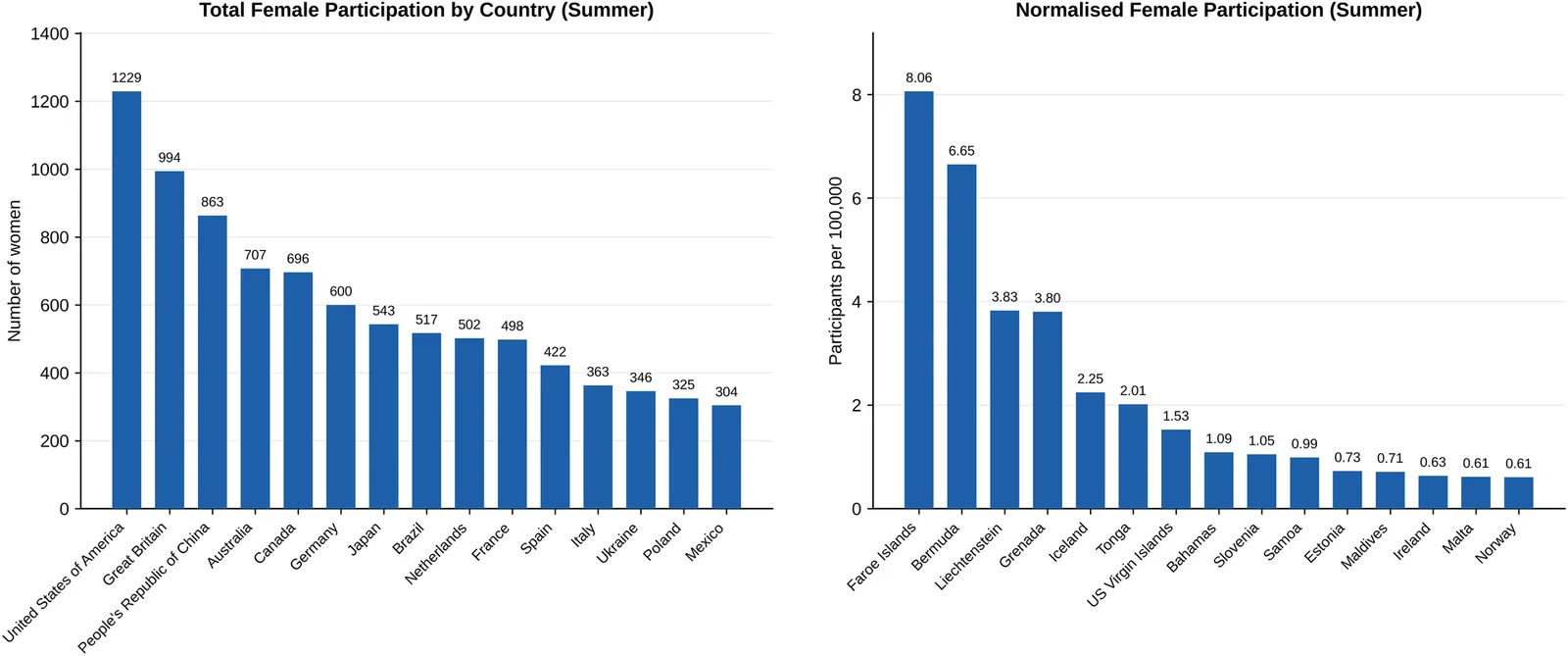
Female participation in Summer Paralympic sports by country. The left panel reports total female participation (absolute values), whereas the right panel shows average female participation per edition, normalized by the female population aged 15–64 and rescaled per 100,000 for readability. The two panels allow comparison between absolute participation levels and the study-specific NFP indicator of demographic-normalized participation intensity across national contexts.

Because absolute participation volumes are strongly influenced by demographic scale and continuity of participation across editions, a demographic-normalized indicator was computed to reassess cross-national differences. When cumulative female participation is adjusted for (a) the number of editions in which each NPC participated and (b) the size of the female population aged 15–64, the ranking changes substantially. Small-population NPCs with sustained Paralympic engagement—most notably the Faroe Islands, Bermuda, and Liechtenstein—exhibit exceptionally high normalized participation intensities, followed closely by Grenada, Iceland, and Tonga. Elevated values are also observed for the US Virgin Islands, Bahamas, Slovenia, and Samoa. Further moderate but still comparatively high intensities appear for Estonia, Maldives, Ireland, Malta, and Norway. Overall, the normalized distribution highlights the prominence of very small NPCs—several of them island states or microstates with only a handful of women athletes entered across a limited number of editions, which contrasts markedly with rankings based solely on absolute cumulative counts (Figure 3; Table 4). As noted in the Methods section, the NFP values for these top-ranked NPCs should be interpreted alongside the underlying raw counts, given the sensitivity of the index to small numerators and small population denominators.

Taken together, the absolute and normalized distributions indicate that long-term patterns of women's participation in the Paralympic Movement reflect both the size of national delegations and the demographic and temporal conditions underpinning *each* NPC's involvement.

#### Distribution by country—Winter Games

The disaggregated analysis of women's participation in the Winter Paralympic Games shows substantially lower absolute volumes than in the Summer Games and a pronounced concentration within a limited group of NPCs. In absolute terms, the United States and Canada have the highest cumulative numbers of female athletes, followed by Austria, Norway, Japan, and Germany—countries with established competitive traditions and infrastructures in winter parasports. Many other NPCs show comparatively small totals and, in numerous cases, irregular or discontinuous participation across editions (Figure 4).

**Figure 4:**
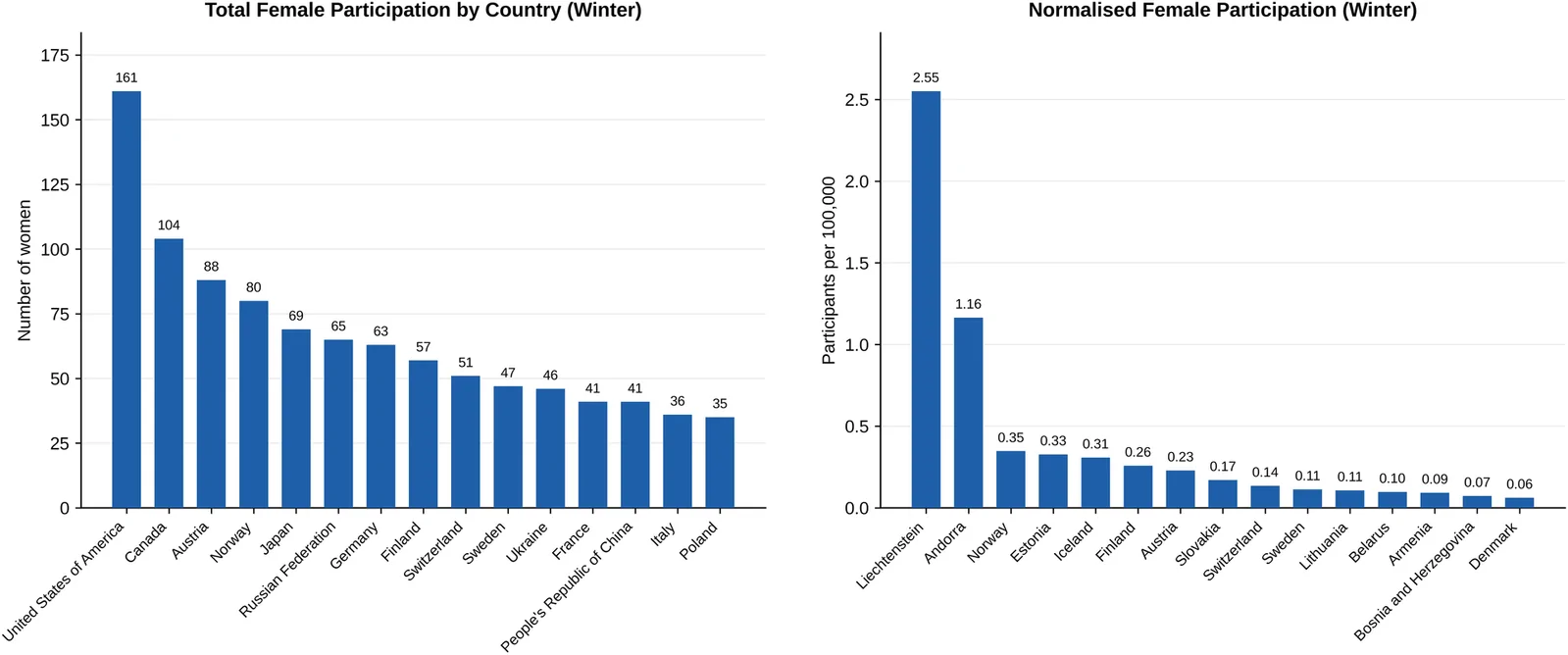
Female participation in Winter Paralympic sports by country. The left panel reports total female participation (absolute values), whereas the right panel shows average female participation per edition, normalized by the female population aged 15–64 and rescaled per 100,000 for readability. The two panels allow comparison between absolute participation levels and the study-specific NFP indicator of demographic-normalized participation intensity across national contexts.

As in the Summer Games, these raw volumes reflect structural factors such as delegation size, demographic scale, and continuity of participation. To account for these effects, the demographic-normalized participation index was also applied to the Winter Games dataset, adjusting cumulative female counts by (a) the number of Winter Paralympic editions in which each NPC participated and (b) the size of the female population aged 15–64.

Once normalized, the cross-national pattern changes substantially. Several NPCs with small populations but sustained participation, particularly Nordic and Alpine microstates and smaller European delegations, display markedly higher participation intensities than large-population countries, whose absolute totals decrease considerably when adjusted for demographic scale. Conversely, major winter-sports nations, such as the United States, Japan, or Germany, show lower normalized values despite high absolute numbers (Figure 4; Table 5). Taken together, the absolute and normalized distributions indicate that women's long-term participation in the Winter Paralympic Games varies widely across NPCs and is strongly shaped by demographic scale and continuity of involvement.

#### Regional differences in normalized female participation

The regional comparison of NFP values revealed significant differences across geographical macro-areas in both the Summer and Winter Paralympic Games (Table 8; Figure 5). For the Summer Games, the Kruskal–Wallis test indicated a significant regional effect [*H*(5) = 47.93, *p* < 0.001]. Western Europe and North America showed the highest median NFP values (0.25 and 0.94, respectively); however, the North America estimate was based on only four NPCs, two of which were small island territories (Bermuda and US Virgin Islands) with disproportionately high demographic-normalized rates, and should therefore be interpreted with the same caution applied below to the Winter Games North America group, whereas Latin America, the Asia-Pacific, and Africa and the Middle East displayed substantially lower median values. Pairwise *post hoc* comparisons with Holm correction showed that Western Europe had significantly higher NFP values than Latin America, the Asia-Pacific, and Africa and the Middle East. Eastern Europe/Former USSR and North America also showed significantly higher NFP values than Africa and the Middle East. Western Europe and North America did not differ significantly.

**Table 8:** Regional distribution of normalized female participation in the Summer and Winter Paralympic Games.

Macro-area	Summer Games, n	Summer Games NFP median	Summer Games IQR	Winter Games, n	Winter Games NFP median	Winter Games IQR
Western Europe	24	0.25	0.42	17	0.06	0.24
Eastern Europe/Former USSR	30	0.13	0.32	23	0.02	0.06
North America	4	0.94	2.53	2	0.04	0.02
Latin America	30	0.04	0.06	5	0.00	0.00
The Asia-Pacific	36	0.02	0.25	7	0.01	0.01
Africa and the Middle East	64	0.01	0.05	4	0.00	0.01

NFP = Normalized Female Participation Index, rescaled per 100,000 female inhabitants aged 15–64. Values are reported as medians and interquartile ranges because distributions were highly skewed. Regional differences were significant for both the Summer Games [Kruskal–Wallis H(5) = 47.93, *p* < .001] and Winter Games [Kruskal–Wallis H(5) = 20.27, *p* = .001]. Pairwise comparisons were conducted using Dunn's test with Holm correction.

**Figure 5:**
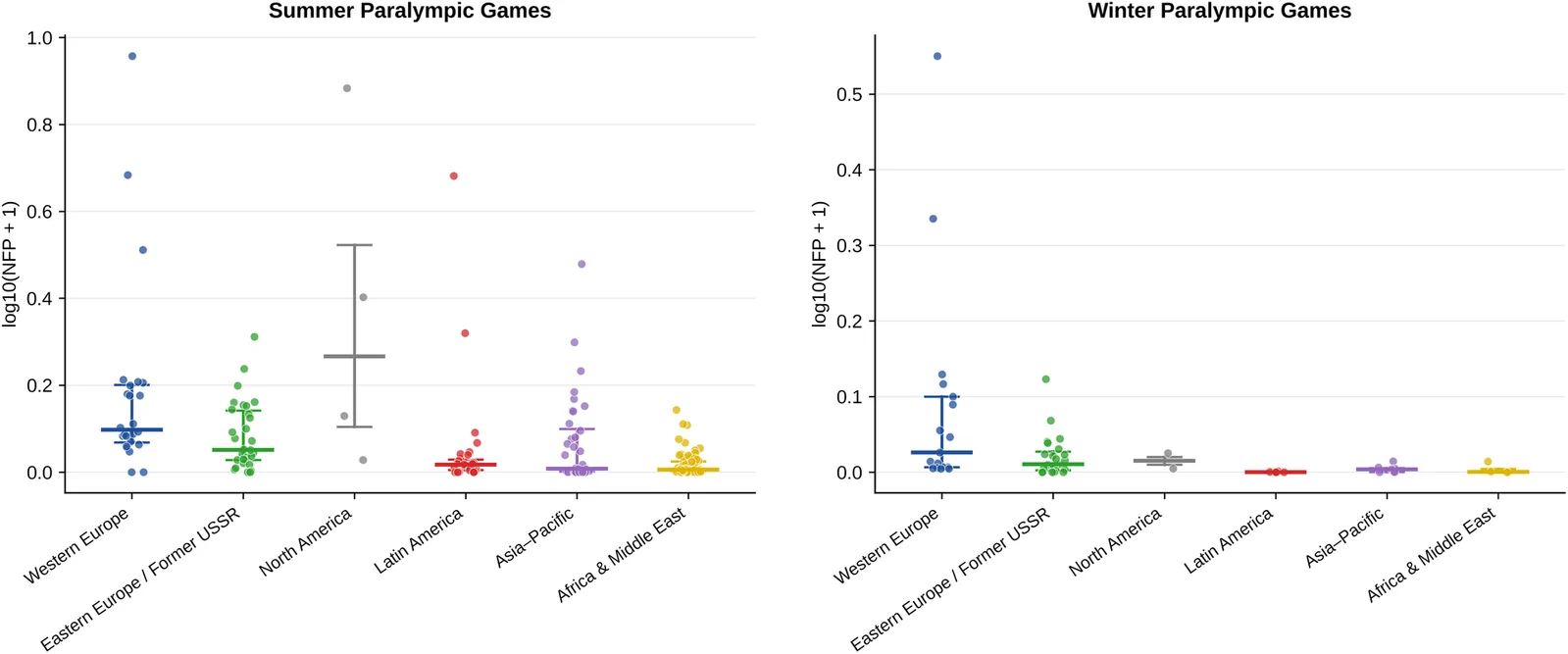
Regional distribution of normalized female participation in the Summer and Winter Paralympic Games. The figure shows country-level NFP values by geographical macro-area for the Summer and Winter Games. Values are displayed on a log10(NFP + 1) scale to improve visualization of highly skewed distributions.

For the Winter Games, regional differences were also significant [*H*(5) = 20.27, *p* = 0.001]. Western Europe again showed the highest median NFP value, followed by North America, although the latter group included only two countries and should therefore be interpreted cautiously. *Post hoc* comparisons indicated that Western Europe had significantly higher NFP values than Latin America and the Asia-Pacific. Overall, these findings indicate that demographic-normalized female participation is unevenly distributed across macro-areas, with Western Europe showing among the highest participation intensity in both Summer and Winter Games.

#### Country-level trajectories of participation across historical cycles

The analysis of country-level time series reveals markedly differentiated trajectories across NPCs in both the Summer and Winter Games. For each NPC and edition, we calculated the proportion of women among the total number of accredited athletes, thus capturing the internal sex composition of national delegations over time, independently of overall delegation size.

Some countries displayed an early and relatively continuous increase in female representation, with progressive convergence toward values approaching parity in the most recent editions. In the Summer Games, the Netherlands, Canada, Australia, and Great Britain show this pattern, generally recording female participant proportions between 30% and 50% from the late 1980s and 1990s onwards, with further increases in the latest cycles (e.g., Canada exceeded 55% in Paris 2024; the Netherlands remained above 50% from 2008 onward). In the Winter Games, relatively stable and non-intermittent patterns emerged in countries such as Finland, Norway, and Canada, where the proportion of women participants rarely fell below 20%–30% across editions from 1994.

A second profile concerns countries characterized by a long initial phase of absence or marginality of female participation, followed by a rapid expansion in recent Paralympic cycles. In the Summer Games, this pattern appeared in countries such as the People's Republic of China, Mexico, Nigeria, Uzbekistan, and Algeria, which showed very low or zero female participation until the 1990s and then stabilized at percentages between roughly 40% and 60% in more recent editions. In the Winter Games, Ukraine, Belarus, and the People's Republic of China displayed a relatively recent entry accompanied by a steep rise, reaching proportions of approximately 25%–50% in the 2014–2022 period.

A third group of countries had intermittent female participation, with alternating editions with a substantial presence and others with no women at all. This pattern is particularly common in the Winter Games and was observed in NPCs with small athlete pools or strong dependence on a few key athletes, such as Bosnia and Herzegovina, Kazakhstan, Croatia, Iceland, Chile, Greece, and Belgium, which often oscillated between 0% and 50%–100% from one edition to the next. In the Summer Games, similar intermittent trajectories were found in several countries with low overall athlete numbers, especially in Africa and Asia.

Finally, a non-negligible set of NPCs showed persistently male-dominant profiles, with no clear signs of convergence over the long term. In the Winter Games, this pattern was evident in nations with established parasports traditions such as France, Italy, Germany, and Japan, where female participant proportions rarely exceed 20%–30% from 1976 to 2022. In the Summer Games, countries such as Egypt, India, Indonesia, and Iraq consistently remained below 25% across the entire historical series.

Overall, these trajectories suggest that the global expansion of women's participation has followed heterogeneous country-level paths, ranging from early and sustained growth to late but rapid increases, intermittent participation, and persistent male dominance (see Supplementary Material 1 for the complete country-level trajectories of internal sex composition across all Paralympic editions).

### Descriptive bibliometric analysis of research on women in Paralympic sports

#### General information on the corpus

The descriptive bibliometric analysis was conducted on a final corpus of 71 publications indexed in the Web of Science Core Collection and selected according to the inclusion criteria detailed in the Methods section (see Supplementary Materials 2 for the full list of included documents and their indexing information). The publications span 1999–2025 and are distributed across 54 distinct sources, with an annual growth rate of 9.66%. The corpus comprises 2,285 references, with an average of 11.97 citations per publication and a mean publication age of 5.33 years, indicating a comparatively recent and rapidly expanding area of research.

With regard to publication typology, the dataset primarily consists of original research articles (*n* = 56), alongside case reports and case studies (*n* = 12) and a small number of reviews (*n* = 3, including one early-access review). Thus, the field is currently dominated by empirical studies focusing on specific cases, interventions, or athletic populations, while systematic syntheses and conceptual reviews remain limited.

In terms of thematic focus, 47 publications (≈66.2%) address the Summer Paralympic Games, 6 contributions (≈8.4%) concern the Winter Paralympic Games, and 18 articles (≈25.3%) deal with the Paralympic Movement more generally, without reference to a specific edition or seasonal classification.

The distribution of sports and activity domains is markedly heterogeneous. In total, 23 studies address Paralympic sports in general, without a single-sport focus. Among specific disciplines, wheelchair basketball appears most frequently (16 publications), followed by track and field (5) and cross-country sit-skiing (4). Other sports are represented by smaller clusters, including para-skiing (1), para-equestrian (2), wheelchair tennis (2), football for athletes with cerebral palsy (2), and para-powerlifting (2). A broader range of disciplines, such as goalball, blind judo, archery, para-ice hockey, sitting volleyball, boccia, para-swimming, and ultra-endurance, are each covered by a single publication (Table 9).

**Table 9:** Overview of the main features of the bibliometric corpus.

Main information about data	Results	Sports analyzed	Results
Timespan	1999–2025	Not specific	23
Sources (Journals, Books, etc.)	54	Wheelchair tennis	2
Documents	71	Wheelchair basketball	16
Annual growth rate %	9.66	Track and field	6
Document average age	5.33	Boccia	1
Average citations per doc	11.97	Football with CP	2
References	2,285	Blind Judo	1
Document types	Results	Para-equestrian	2
Article	55	Cross-country sit-skiing	4
Article; early access	1	Archery	1
Case-report; case-study	12	Goal ball	1
Review	2	Para-ice hockey	1
Review; early access	1	Para-powerlifting	2
TOPIC		Sitting volleyball	4
Summer Paralympic Games	47	Para-skiing	1
Winter Paralympic Games	6	Para-swimming	3
Paralympic Movement	18	Ultra-endurance	1

The table presents key descriptive metrics of the analyzed publications, i.e., time span, sources, document types, thematic distribution, and sports-specific coverage, offering a comprehensive profile of the scientific output on women in Paralympic sports across the period 1999–2025.

Taken together, these indicators depict a small but growing scientific domain, characterized by recent consolidation, a marked predominance of Summer Games-related work, and a highly fragmented distribution of single-sport studies across a wide range of Paralympic disciplines.

#### Sources, affiliations, and thematic keywords

The corpus was distributed across 54 publication sources, indicating a fragmented rather than journal-centered field. The most represented source was Frontiers in Sports and Active Living (four articles), followed by Qualitative Research in Sport, Exercise, and Health and Scientific Reports (three articles each). A further group of journals contributed smaller numbers of articles, including *Adapted Physical Activity Quarterly*, *BMC Sports Science, Medicine and Rehabilitation*, *Journal of Sports Sciences*, and *Research in Sports Medicine*. This distribution suggests that research on women in Paralympic and parasports contexts has not yet consolidated around a single specialist publication source.

The analysis of institutional affiliations showed a similar pattern of partial concentration within a broader fragmented field. The most represented institutions included the University of Tsukuba, Loughborough University, the University of Basque Country, Bournemouth University, Griffith University, Ontario Tech University, Universidad Politécnica de Madrid, and the University of British Columbia. These results suggest the presence of a small number of recurrent research centers, alongside a wider set of institutions contributing occasionally to the field.

The keyword analysis provided an exploratory indication of the thematic structure of the corpus. In Keywords Plus, the most frequent terms included “performance,” “disability,” “women,” “participation,” “sports,” and “gender.” Author keywords showed a partly similar structure, with “disability,” “paralympics,” “women,” “sport,” “gender,” “performance,” “paralympic sport,” “inclusion,” and “parasport” among the most frequent terms. Taken together, these terms suggest that the literature is organized around the following three main thematic areas: performance and sports-specific research; disability and Paralympic participation; and gender, inclusion, and women's experiences in parasports.

Trend-topic outputs should be interpreted cautiously because of the small size of the corpus. Nevertheless, they suggest a recent consolidation of terms related to participation, gender, and disability. Whereas “performance” appears as a more established term in the corpus, terms such as “gender,” “participation,” and “disability” appear more prominently in recent years. This pattern is consistent with a field that has progressively expanded from performance-oriented work toward broader questions of participation, inclusion, and gendered experiences in Paralympic and parasports contexts.

#### Temporal evolution of scientific production (1999–2025)

Over time, scientific output focused specifically on women in the Paralympic and parasports context appears relatively late and follows a progressively increasing trajectory. The earliest article in the corpus dates to 1999, and during the first decade (late 1990s–2000s), production remained limited, with only a few scattered contributions and several years with one or no indexed publications (Figure 6).

**Figure 6:**
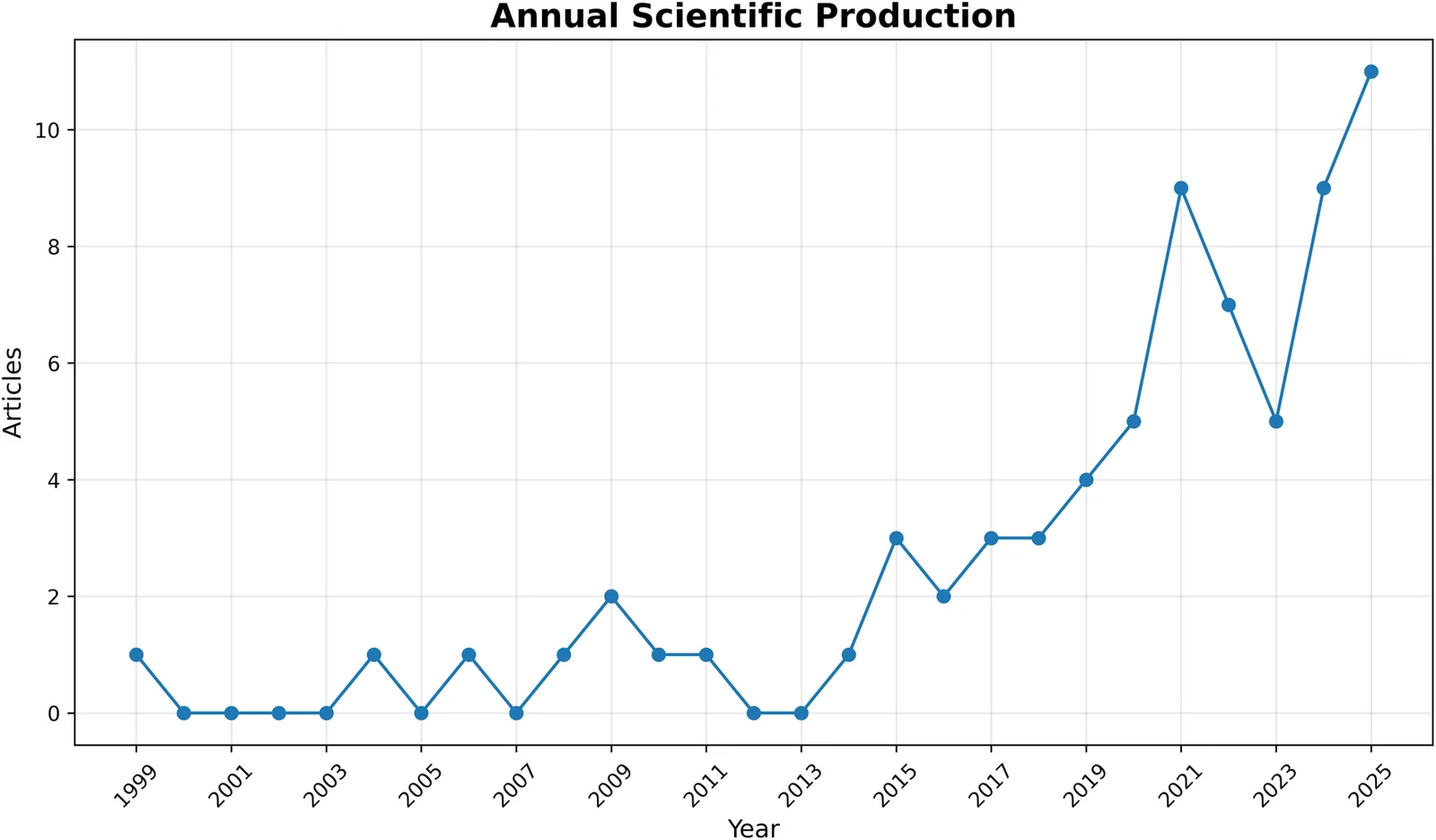
Annual scientific production on women in Paralympic sports (1999–2025). The figure illustrates the annual number of publications included in the final bibliometric corpus over the observed period.

From the 2010s onward, particularly after the London 2012 Paralympic Games, the publication density increases, with a more regular annual output and a steady presence of new contributions through 2025. Overall, the temporal profile shows (a) an initial phase of low productivity and limited continuity (1999 to the late 2000s) and (b) a subsequent phase of consolidation and growth over the past 10–15 years.

#### Geographical distribution of scientific production (affiliation countries)

The mapping of publication output by country of institutional affiliation shows a marked geographical concentration in the scientific production on women in the Paralympic Games. The most productive countries are Spain (10 articles), Great Britain (9), Canada (7), United States (7), Brazil (6), and Japan (6) (Figure 7). Additional European countries contribute smaller but notable volumes, while a limited number of articles originate from emerging research contexts. Overall, the distribution reflects a predominance of institutions based in Western Europe and North America, with Spain and Japan standing out as particularly active contributors.

**Figure 7:**
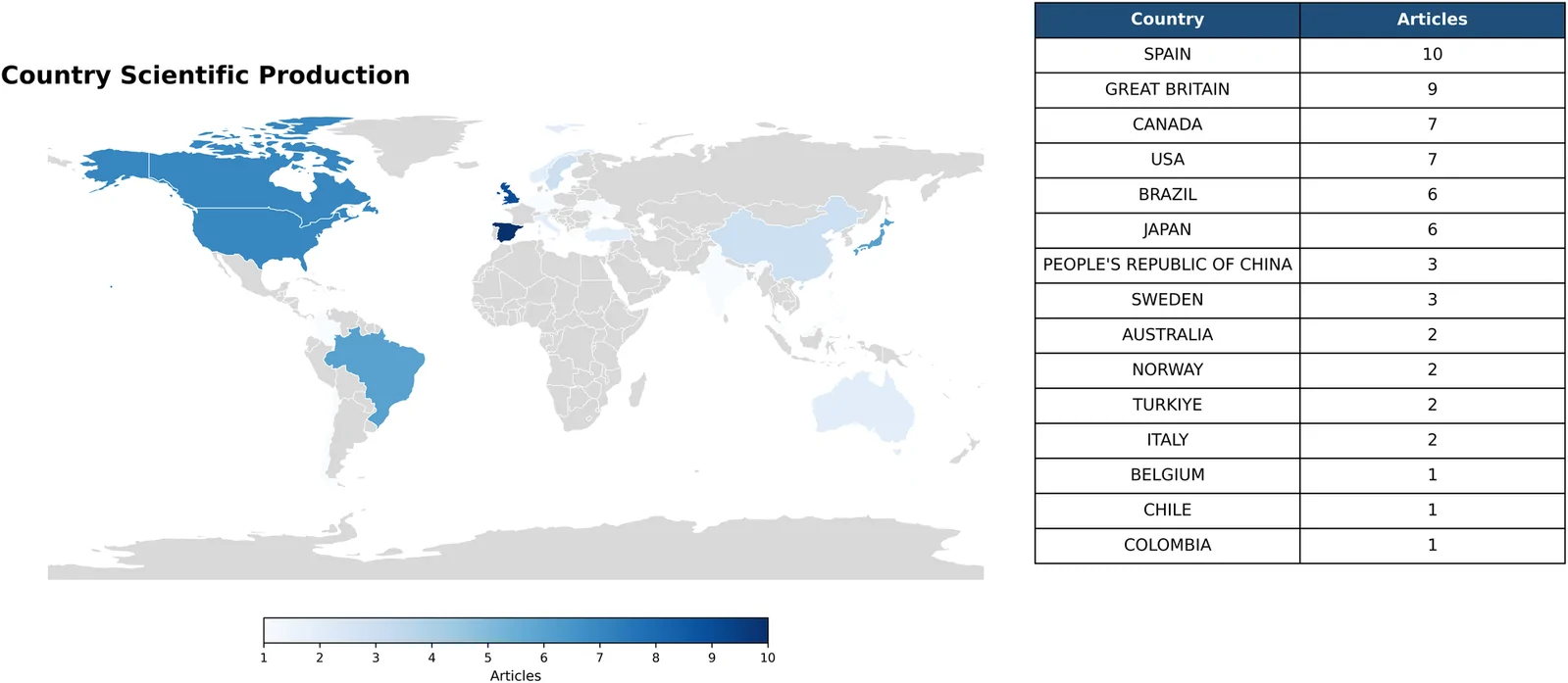
Geographical distribution and leading countries of scientific production on women in Paralympic sports (1999–2025). The figure integrates a world map showing institutional affiliation by volume of publications, with darker shades indicating higher scientific output, and a summary table identifying the countries with the highest number of publications in the analyzed bibliometric corpus.

Historical publication trajectories by affiliation country indicate that most highly productive nations began publishing on this topic systematically only in the last two decades, with output increasing progressively over time.

### Relationship between female participation and scientific production

The distribution of scientific production mirrors the asymmetries observed in participation between the Summer and Winter Paralympic Games. In the Summer Games context, where recent editions show female participant proportions of approximately 40%–45%, the majority of publications in the corpus, 46 out of 71 (about two-thirds), focus explicitly on the Summer Games. These articles cover a wide range of topics, including participation, performance, functional classification, health, media coverage, and the organizational aspects of the Paralympic Movement.

By contrast, the Winter Paralympic Games show both lower female participation and limited academic output. As noted above in *Historical trends—Winter Paralympic Games*, women have consistently represented approximately 20%–25% of Winter Games participants across nearly five decades, with little variation over time. Correspondingly, only six publications in the corpus (less than 10%) made the Winter Games their primary focus, indicating a substantially smaller body of research related to this event.

A further group of 18 articles (≈25.7%) addresses the Paralympic Movement more broadly, without reference to specific editions or seasonal classifications. These contributions are primarily conceptual, sociological, or institutional in scope and examine themes such as governance, inclusion, equity, communication, and media representations. Although they do not provide edition-specific participation data, they constitute a general analytical framework for understanding the patterns observed in the historical series. Overall, the corpus is structured around three main areas, namely, Summer Games-focused studies, Winter Games-focused studies, and broader conceptual analyses, each reflecting distinct orientations in scientific production related to women athletes in Paralympic and parasports contexts.

#### Geographical alignment between female participation and scientific production

Using the macro-regional classification adopted in the previous section, the comparison between participation data and scientific production reveals a pronounced spatial asymmetry. Western Europe, North America, and the Asia-Pacific emerge as the three regions with the highest historical volumes of female participation and the greatest concentration of scholarly output, whereas Africa and the Middle East, Latin America, and Eastern Europe/Former USSR are less represented in both respects.

Regarding scientific production, Western Europe constitutes the principal academic hub, with 32 publications (45.71% of the corpus), followed by North America and the Asia-Pacific with 14 articles each (20%). Latin America contributes eight articles (11.43%), while Africa and the Middle East and Eastern Europe/Former USSR show very limited output [two and one publication(s), respectively]. No publications are associated with the special categories (Unified Team, Independent Paralympic Participants, Individual Paralympic Athletes, and Neutral Paralympic Athletes).

The comparison between these dimensions highlights several patterns. In regions with consolidated Paralympic Games infrastructures, particularly Western Europe and North America, high levels of female participation are accompanied by high research productivity. Other regions display partial mismatches: Latin America and Eastern Europe/Former USSR, for example, register non-negligible historical volumes of female participation but relatively few publications. In the Winter Games, further divergences appear, as some academically productive regions (notably Western Europe and parts of the Asia-Pacific) show comparatively limited or stagnating female representation.

Overall, the macro-regional analysis indicates that the relationship between female participation and scientific production varies across regions, with areas of alignment and misalignment shaping a differentiated global landscape (Table 10).

**Table 10:** Female participation in the Paralympic Games and corresponding scientific production by geographical macro-area.

Geographical macro-area	Total number of athletes—Summer Games (1960–2024)	Total number of athletes—Winter Games (1976–2022)	Total scientific production	Percentage of articles relative to the total corpus (*N* = 71)
Western Europe	6,012	687	32	45.71%
Eastern Europe/Former USSR	749	147	1	1.43%
North America	1,938	265	14	20.00%
Latin America	1,344	3	8	11.43%
Asia-Pacific	2,960	142	14	20.00%
Africa and the Middle East	1,293	5	2	2.86%
Special categories	56	23	0	0.00%

The table summarizes, for each macro-area, the total number of women who competed in the Summer (1960–2024) and Winter Paralympic Games (1976–2022), together with the volume of scientific publications produced by institutions located in the same regions and their share relative to the total bibliometric corpus (*N* = 71). Special categories include the four official IPC non-national categories (Unified Team, Independent Paralympic Participants, Individual Paralympic Athletes, Neutral Para Athlete); historically dissolved national teams (Czechoslovakia, East Germany, West Germany, Yugoslavia, Serbia and Montenegro, and Rhodesia) are assigned to their corresponding geographical macro-area.

#### Country-level scientific production and the WEI

To analyze the relationship between women's participation and national scientific output, we used the WEI, as defined and calculated in the Materials and methods section. Crossing WEI values with scientific production (measured through the institutional affiliations of the articles in the corpus) yields the following four empirical profiles:

1. High WEI and high scientific production
  This group comprises countries that combine high WEI values, reflecting a higher average female share within delegations, with substantial research output, namely, the People's Republic of China (WEI = 41.76; three articles), Canada (WEI = 37.19; seven articles), Norway (WEI = 36.97; two articles), the United States (WEI = 34.08; seven articles), Great Britain (WEI = 34.07; nine articles), and Australia (WEI = 31.59; two articles). These countries show consistently high WEI scores (above 30) alongside sustained publication activity, often involving recurrent institutional affiliations.
2. High scientific production but medium or low WEI
  A second group includes countries with notable research output but less balanced patterns of women's participation, namely, Turkey (WEI = 29.56; two articles), Japan (WEI = 27.39; six articles), Sweden (WEI = 27.32; three articles), Italy (WEI = 26.64; two articles), Spain (WEI = 25.89; 10 articles), and Brazil (WEI = 23.99; six articles). All contribute significantly to the corpus, while their WEI values remain lower than those observed in the first group.
3. High WEI but low scientific production
  A third group brings together countries characterized by high WEI values and comparatively limited publication activity, namely, the Philippines (WEI = 39.03; one article), Ireland (WEI = 38.79; one article), the Netherlands (WEI = 38.22; one article), Ukraine (WEI = 35.23; one article), Germany (WEI = 33.27; one article), and the Czech Republic (WEI = 32.14; one article). These countries reach some of the highest WEI scores in the dataset (all above 32) but are only marginally represented in the scientific corpus, each associated with a single publication.
4. Medium or low WEI and low scientific production
  The remaining countries form a more heterogeneous group with lower levels of both WEI and research output, including Chile (WEI = 23.09; one article), Colombia (WEI = 21.18; one article), Belgium (WEI = 19.36; one article), India (WEI = 16.18; one article), Greece (WEI = 15.33; one article), and Malaysia (WEI = 13.5; one article).

Overall, the joint distribution of WEI and scientific output delineates a differentiated landscape in which countries occupy distinct quadrants according to their internal delegation gender balance and level of publication activity (Table 11; Figure 8).

**Table 11:** Scientific production and WEI by country.

Country	Number of articles	Summer Games WEI	Winter Games WEI
Spain	10	25.9	24.2
Great Britain	9	34.1	15.4
Canada	7	37.2	25.7
USA	7	34.1	25.5
Brazil	6	24.0	16.7
Japan	6	27.4	17.5
People's Republic of China	3	41.8	26.4
Sweden	3	27.3	20.4
Australia	2	31.6	10.4
Norway	2	37.0	21.2
Turkey	2	29.6	—
Italy	2	26.6	10.7
Belgium	1	19.4	30.0
Chile	1	23.1	4.2
Colombia	1	21.2	—
Czech Republic	1	32.1	18.4
Germany	1	33.3	29.5
Greece	1	15.3	20.0
India	1	16.2	—
Ireland	1	38.8	—
Malaysia	1	13.5	—
Netherlands	1	38.2	28.1
Philippines	1	39.0	—
Ukraine	1	35.2	40.8

The table reports, for each country represented in the bibliometric corpus (*N* = 71 articles), the number of publications associated with national institutional affiliations and the corresponding WEI for the Summer and Winter Paralympic Games. The WEI expresses a normalized measure of within-country sex balance across Paralympic cycles, combining the proportion of women in national delegations with the continuity of their participation over time.

**Figure 8:**
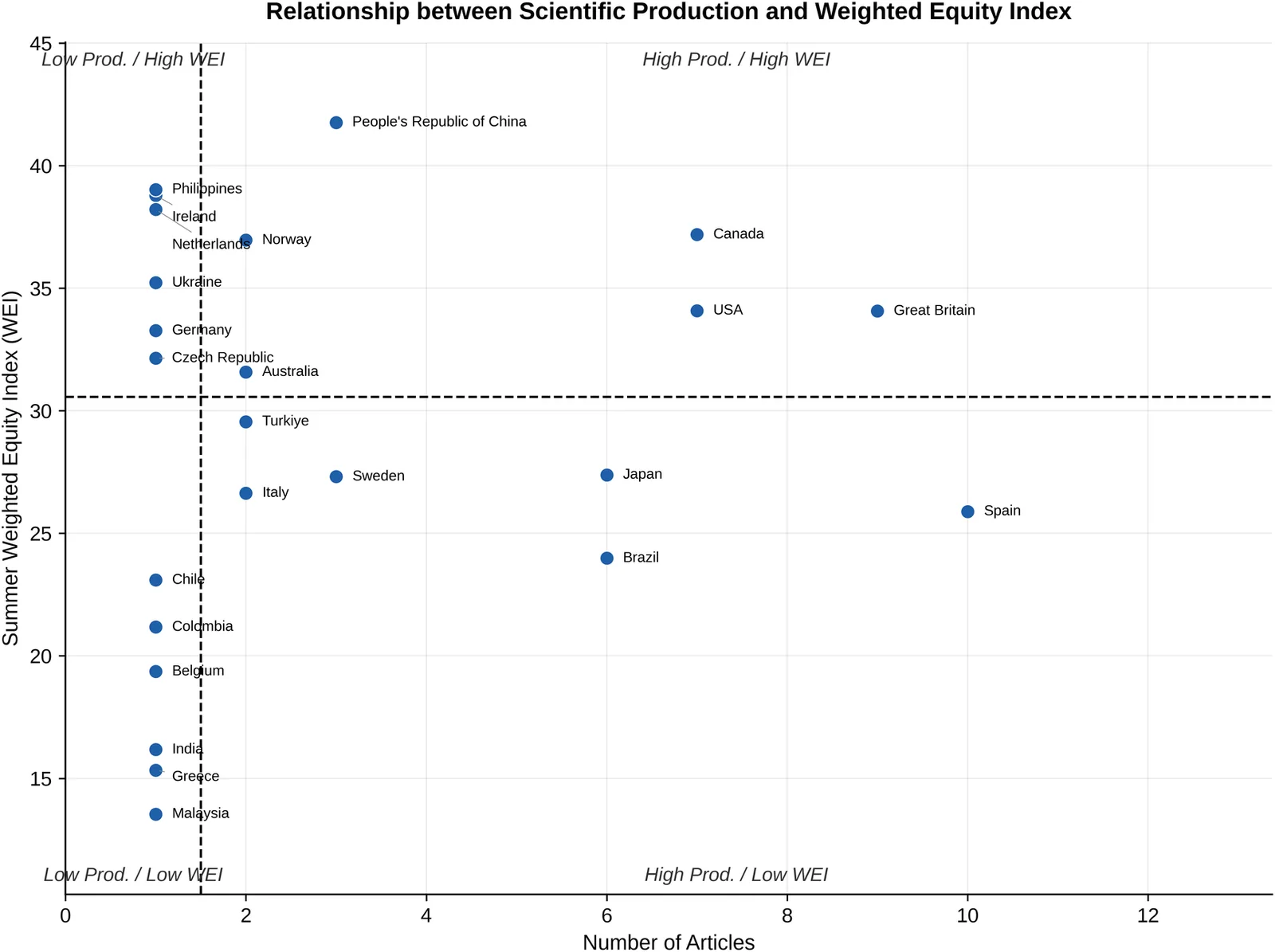
Relationship between national scientific production and the Summer Games WEI. The scatterplot illustrates the association between the number of scientific publications produced by each country and its Summer Games WEI, a study-specific descriptive indicator summarizing the average female participant proportion within Summer Paralympic Games delegations across participating editions. Vertical and horizontal dashed lines represent the median values of scientific production and WEI, respectively, delimiting four analytical quadrants that differentiate countries with high/low production and high/low WEI values.

The same classification, applied to the Winter Games WEI values reported in Table 11 (median articles = 2; median Winter Games WEI = 20.80), yields an analogous four-group structure, as follows:

1. High WEI and high scientific production
  This group comprises the People's Republic of China (WEI = 26.45; three articles), Canada (WEI = 25.67; seven articles), the United States (WEI = 25.50; seven articles), Spain (WEI = 24.17; 10 articles), and Norway (WEI = 21.22; two articles). Four of these five countries—China, Canada, the United States, and Norway—also occupy this quadrant in the Summer Games analysis, indicating a consistent alignment between internal delegation gender balance and scientific visibility across both types of Games.
2. High scientific production but medium or low WEI
  This group includes Sweden (WEI = 20.37; three articles), Japan (WEI = 17.51; six articles), Brazil (WEI = 16.67; six articles), Great Britain (WEI = 15.43; nine articles), Italy (WEI = 10.74; two articles), and Australia (WEI = 10.41; two articles). Sweden, Japan, Brazil, and Italy occupy the same quadrant in the Summer Games analysis. Great Britain and Australia, by contrast, are positioned in the high WEI/high production quadrant in the Summer Games analysis and shift into this lower-WEI group in the Winter Games analysis.
3. High WEI but low scientific production
  This group comprises Ukraine (WEI = 40.83; one article), Belgium (WEI = 30.00; one article), Germany (WEI = 29.53; one article), and the Netherlands (WEI = 28.12; one article). Germany, the Netherlands, and Ukraine also occupy this quadrant in the Summer Games analysis, each associated with a single publication despite ranking among the highest WEI scores in both events; Belgium, which falls into the lowest-WEI group in the Summer Games analysis, reaches this quadrant only in the Winter Games analysis.
4. Medium or low WEI and low scientific production
  This group includes Greece (WEI = 20.00; one article), the Czech Republic (WEI = 18.37; one article), and Chile (WEI = 4.17; one article). Greece and Chile occupy the same quadrant in the Summer Games analysis, whereas the Czech Republic is positioned in the high WEI/low production quadrant in the Summer Games analysis and moves into this group in the Winter Games analysis.

Overall, the Winter Games WEI shows the same broad structure as the Summer Games WEI, with a subset of countries—the People's Republic of China, Canada, the United States, Norway, Germany, the Netherlands, and Ukraine—maintaining a stable position across both events, while a smaller number of countries (Great Britain, Australia, Spain, Belgium, and the Czech Republic) shift quadrant between the Summer and Winter Games, generally reflecting the lower and more volatile WEI values observed for the Winter Games (Table 11; Figure 9) (see also the note on small-sample sensitivity in the Materials and methods section).

**Figure 9:**
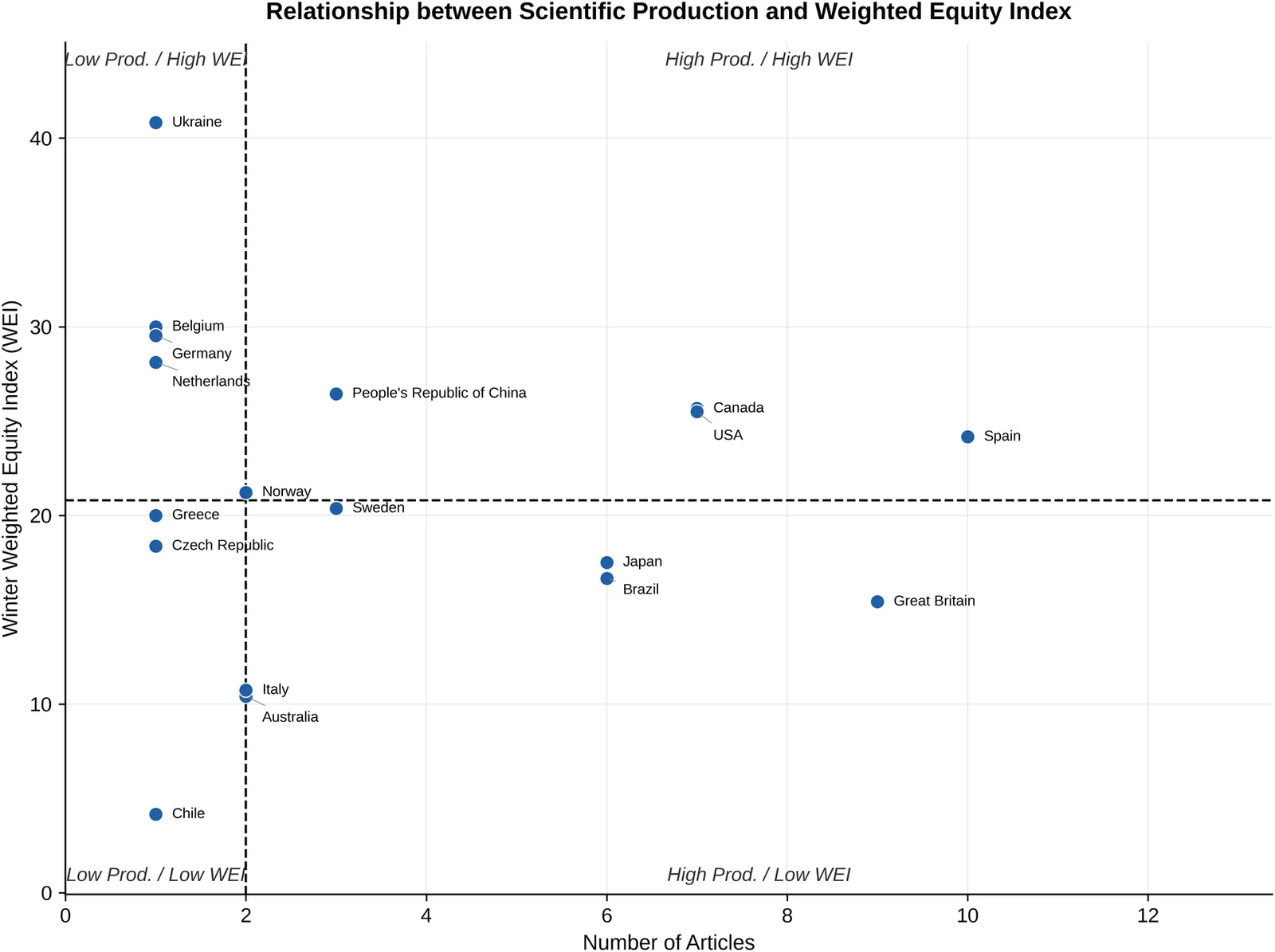
Relationship between national scientific production and the Winter Games WEI. The scatterplot displays the association between the number of scientific publications produced by each country and its Winter Games WEI, a study-specific descriptive indicator summarizing the average female participant proportion within Winter Paralympic Games delegations across participating editions. Vertical and horizontal dashed lines represent the median values of scientific production and WEI, respectively, delimiting four analytical quadrants that differentiate countries with high/low production and high/low WEI values.

## Discussion

In this section, we discuss the main empirical patterns emerging from the analysis of women's participation in the Paralympic Games and the related scientific production. We focus on the historical evolution of participation, the diversity of national trajectories, the characteristics of the bibliometric corpus, and the convergence or divergence between competitive opportunities and scholarly attention.

### Historical evolution of women's participation: Summer Games convergence and limited Winter Games growth

Women's participation shows divergent trajectories in the Summer and Winter Paralympic Games. In the Summer Games, representation increases in the 1960s–early 1970s, stagnates from the late 1970s to the 1990s, and then rises sharply from the 2000s, reaching near parity in recent editions. In contrast, Winter Games participation shows only a modest increase over time and remains within a persistently lower range, approximately one-fifth to one-quarter of the athletes, with no comparable movement toward parity. These patterns are consistent with non-linear, season-specific pathways that may be influenced by infrastructure, the diffusion of disciplines, and national programs. WEI and NFP were used to complement the calculation of aggregate female participant proportions by summarizing cross-national differences in internal delegation balance and demographic-normalized participation intensity.

### National profiles and new indicators (WEI and NFP)

The country-level analysis suggests that the overall increase in women's participation includes at least four recurrent trajectories: early and sustained increase, late but rapid growth, intermittent participation, or a persistently male-dominant profile. These contrasts appear particularly pronounced in the Winter Games, where the smaller participation base and the geographical concentration of snow and ice disciplines may contribute to the difference between countries with relatively continuous female involvement and those with fragmented or minimal engagement.

In this perspective, the WEI provides a descriptive summary of the average female participant proportion within each NPC's delegation across participating editions. It helps distinguish NPCs with consistently higher internal female representation from those with lower average delegation gender balance over time.

The fact that some NPCs with relatively small delegations achieve high WEI scores suggests that delegation gender balance is not reducible to the overall size of national sports systems. It may also be associated with organizational priorities, recruitment pathways, and support structures for female athletes, consistent with qualitative evidence that national Paralympic Games success is shaped less by aggregate structural variables than by the coherence of specific policy interventions, including athlete classification frameworks and the integration of Paralympic-specific knowledge into national sports systems (29). Comparative evidence from Latin America shows that the promotion of Paralympic sports depends on a country's capacity to build accessible, continuous, and equitable systems of training and support, rather than on incentive volume or medal performance alone (30).

The NFP complements this view by showing how cross-national comparisons change once demographic size and continuity of engagement are taken into account. When participation is examined through this demographic-adjusted lens, small-population NPCs with consistent Paralympic involvement move to the top of the rankings, whereas some large countries with high absolute numbers of women rank lower. This pattern points to forms of participation intensity that would remain less visible if the analysis relied solely on raw delegation sizes, and is broadly consistent with evidence that country-level Paralympic participation is shaped by economic factors independent of population size, with this association found to be strongest specifically for female athletes (31). As noted in the Materials and methods section, however, NFP values are sensitive to small numerators and small population denominators, so the positioning of small-population NPCs at the top of the ranking should be read as an indication of participation intensity rather than as a stable or precisely estimated quantity.

Taken together, WEI and NFP help differentiate how women's participation can be described: not only through the number of female athletes sent to the Games, but also through the average internal gender balance of delegations, the continuity of women's participation, and participation density relative to the female population.

### Characteristics of scientific production and geographical imbalances

The bibliometric analysis identifies a small but growing corpus (71 articles, 1999–2025), largely focused on the Summer Paralympics and summer disciplines, with limited focus on the Winter Games. Output was sparse until the early 2010s, then increased steadily over the last decade, particularly after London 2012. This temporal pattern is consistent with the relatively recent consolidation of women in Paralympic and parasports contexts as a specific object of scientific attention.

The additional bibliometric outputs refine this picture. The distribution of articles across 54 publication sources suggests a fragmented rather than journal-centered field, with no single specialist source clearly organizing the literature, a pattern broadly consistent with bibliometric mapping of Olympic sports science more generally, where research output is similarly dispersed across a wide publication base rather than concentrated in a small number of specialist outlets (15). A similar pattern emerges at the level of institutional affiliations: a small number of universities recur more frequently, but the field remains distributed across a wider set of institutions contributing smaller numbers of publications. This suggests that research on women in Paralympic and parasports contexts is growing, but has not yet developed a strongly consolidated institutional or editorial infrastructure.

The keyword and trend-topic outputs also suggest a field in transition. Terms related to performance and sports-specific research remain central, but more recent terms such as gender, participation, disability, and inclusion point toward a gradual broadening of the research agenda. This should be interpreted cautiously, given the small size of the corpus, but it is consistent with a movement from primarily performance-oriented studies towards a more diversified literature addressing participation, representation, inclusion, and athletes' experiences, echoing broader calls within women's sports research to move beyond narrow performance metrics and engage more directly with structural and experiential dimensions of participation (14).

Geographically, production remains concentrated in a few countries—mainly in Western Europe, North America, and the Asia-Pacific (notably Spain, the United States, Great Britain, Canada, and Japan)—while Africa, the Middle East, and Eastern Europe/former USSR are underrepresented. This distribution may be related to differences in research infrastructures, funding opportunities, international academic networks, and the dominance of English-language publishing. It should therefore be interpreted not only as a map of scientific interest, but also as a map shaped by indexing, language, and institutional visibility, consistent with documented biases in the journal coverage of Web of Science itself, which disproportionately indexes English-language publications and journals based in Western Europe and North America relative to global scholarly output (32).

### Alignments and divergences between participation and scientific production

The descriptive comparison between participation and bibliometric data suggests both alignments and mismatches between women's competitive presence and scholarly research. At a macro-regional level, Western Europe, North America, and the Asia-Pacific tend to combine relatively higher women's participation with higher scientific output, whereas other regions remain less visible in the literature despite documented participation growth. The WEI–publication quadrant analysis was used to organize countries into four descriptive profiles: high WEI/high output; high output with medium or low WEI; high WEI/low output; and medium or low values in both dimensions. Overall, participation gains do not appear to translate automatically into academic visibility, or vice versa. This misalignment may be associated with differences in research infrastructure and funding, the presence of specialized research groups, the priorities of federations and NPCs, and access to international scientific networks. This is consistent with evidence that sports science research institutions remain heavily concentrated in a small number of countries, with the most highly ranked sports science universities clustered almost exclusively in North America, the United Kingdom, and a handful of European nations (33), and the priorities of federations and NPCs, which shape not only competitive outcomes but also the institutional visibility of national Paralympic programs (29).

### Implications for sports policies and the research agenda

From a sports policy perspective, the Summer Games female participation trajectory suggests that movement toward parity may be supported by long-term strategies, including expansion of the event program, dedicated funding, youth development, and structured high-performance pathways, consistent with qualitative evidence that Paralympic success is shaped by the coherence of specific national policy interventions rather than by aggregate structural variables alone (29). The countries with high WEI and NFP values suggest that some demographically small countries have developed relatively strong and continuous patterns of women's participation. However, these indicators cannot by themselves identify the organizational mechanisms behind such patterns, and, as noted above, the corresponding rankings should be interpreted with particular caution where they rest on a small number of editions or a small population base. By contrast, the persistent gap in the Winter Games points to an area requiring further attention. This may be linked to infrastructure costs, geographical constraints, and the more limited diffusion of winter sports, which are themselves expected to narrow further as climate change reduces the number of locations capable of reliably hosting snow- and ice-based competition (34). Targeted measures, such as regional development programs, incentives for women athletes, and partnerships between NPCs, could be explored as possible ways to reduce these imbalances.

From a future research standpoint, the bibliometric mapping points to the following three directions: strengthening work on the Winter Games and under-studied disciplines; expanding collaborations with academic institutions in underrepresented regions; and integrating quantitative and qualitative approaches, such as interviews, ethnographies, and case studies, in line with recent calls to complement participation statistics with in-depth accounts of athletes' experiences and the organizational contexts that shape gender equity in disability sport (13), to better understand how policies, sports cultures, and athletes' biographies may contribute to the observed patterns.

### Strengths and limitations

A main strength of this study is that it provides, to our knowledge, the first historical reconstruction of women's participation across the full trajectory of both the Summer and Winter Paralympic Games, covering the period from Rome 1960 to Paris 2024 for the Summer Games and from Örnsköldsvik 1976 to Beijing 2022 for the Winter Games. By considering both Games within a single framework, the study extends previous work mainly focused on the modern Summer Paralympic period and allows the different temporal trajectories of Summer and Winter Games participation to be examined comparatively.

A second strength is the integrated design of the study, which combines historical participation data with a bibliometric mapping of scientific production on women in Paralympic and parasports contexts. This approach makes it possible to examine not only how women's participation has changed over time, but also whether scholarly attention has developed in parallel with, or separately from, these participation patterns.

Finally, the study is based on a harmonized dataset constructed from IPC archival sources and introduces two clearly defined and reproducible descriptive indicators, the WEI and the NFP. These indicators do not replace conventional measures such as absolute counts and participation percentages, but provide complementary perspectives on internal delegation gender balance and demographic-normalized participation intensity.

This study has some limitations that should be acknowledged. First, participation data were drawn from IPC archival sources, whose completeness and consistency vary across editions. As a partial, targeted check on data reliability, female participation figures for two editions—Rio 2016 and Beijing 2022—were compared against independent tallies published by the Women's Sports Foundation (10, 11), which compiles Olympic and Paralympic participation data from sources separate from the present study's own IPC-based reconstruction. Agreement was close for Rio 2016 [1,669 women and 2,648 men reported by Houghton, Pieper, and Smith (11) against 1,671 and 2,657 in the present dataset]. For Beijing 2022, a larger discrepancy emerged [148 women and 440 men reported by Houghton, Pieper, and Smith (10), against 136 and 422 in the present dataset], plausibly reflecting differences between start-list and final-results counts for an edition marked by an unusually high number of late COVID-19-related withdrawals. This spot-check supports the general reliability of the IPC-based reconstruction while illustrating that even official participation figures can vary depending on the reporting snapshot used, and it should not be read as a systematic validation of the full 1960–2024 series. This issue is particularly relevant for the earlier Paralympic Games, where sex-disaggregated documentation may be less complete and less standardized than in recent editions, as quantified above in *Documentary sources and participation data*. Although data-cleaning and harmonization procedures were applied, these archival differences may affect longitudinal comparability and may lead to partial reconstruction of women's participation in some early editions.

Second, the analysis relies on the sex categories reported in the official IPC records. This was necessary for historical consistency, but it means that the study cannot address gender identity, non-binary participation, or broader gendered experiences within Paralympic sports. The findings should therefore be interpreted as sex-disaggregated participation patterns rather than as a full account of gender equity.

Third, the bibliometric component was based exclusively on English-language publications indexed in the Web of Science Core Collection. This choice ensured consistency and replicability, but it may have excluded relevant work published in other languages, in non-indexed journals, books, reports, theses, or policy documents, consistent with documented evidence that Web of Science journal coverage is itself skewed toward English-language publications (32). Moreover, scientific production was assigned according to authors' institutional affiliations, which may overrepresent countries with stronger academic infrastructures and underrepresent regions where relevant research or athlete participation exists but is less visible in indexed international journals.

Finally, the WEI and NFP are study-specific descriptive indicators derived from observed participation counts. They should not be interpreted as externally validated measures of gender equity, policy effectiveness, or access to Paralympic pathways. The WEI summarizes internal delegation gender balance only across editions in which an NPC participated, while the NFP uses the female population aged 15–64 as a proxy denominator because comparable historical data on women with disabilities are not available across countries and editions. As noted in the Materials and methods section, both indices are additionally sensitive to the number of editions or the size of the population base on which they are computed, so rankings involving NPCs with few participating editions, small delegations, or small national populations should be interpreted as indicative of participation intensity rather than as precise or stable estimates. Future research should refine these indicators and combine longitudinal participation data with qualitative and policy-oriented approaches to better understand the mechanisms behind the observed patterns.

## Conclusion

This study reconstructed women's Paralympic participation across the Summer Games (1960–2024) and Winter Games (1976–2022) and examined these patterns in relation to the geography of scientific production on women in Paralympic sports. Women's participation has increased markedly in the Summer Games, approaching parity in recent editions, whereas participation in the Winter Games remains persistently lower, with only modest long-term growth and no comparable movement toward parity. Country-level patterns, summarized through the study-specific WEI and NFP indicators, show marked variation in internal delegation gender balance and demographic-normalized participation intensity. The bibliometric results indicate that research on Paralympic women is still limited, regionally uneven, and concentrated mainly on the Summer Games and a limited set of sports and themes. Overall, participation and scientific knowledge appear to have developed unevenly across countries, regions, and Games. These patterns point to areas for further research and policy attention, including women's participation in the Winter Paralympic Games, the expansion of research across underrepresented regions and disciplines, and the continued refinement of descriptive indicators for monitoring participation equity.

## Data Availability

The original contributions presented in the study are included in the article/Supplementary Material, further inquiries can be directed to the corresponding author/s.

## Footnotes

^1^In this article, we adopt the widely used distinction between sex and gender in health and social science research. Sex refers to biological characteristics (e.g., chromosomes, hormones, and reproductive anatomy), whereas gender denotes socially constructed roles, norms, and identities. Because this study relies on sex-disaggregated data provided by the International Paralympic Committee, the term *sex* is used when referring to measured variables, while *gender* is employed when addressing broader social and structural issues. In line with current inclusive language guidelines, we use “women” when referring to adult participants or to the relevant social group, while “female” is used adjectivally (e.g., “female athletes”) when referring to sex-based classification (4–9).
